# Text mining to support abstract screening for knowledge syntheses: a semi-automated workflow

**DOI:** 10.1186/s13643-021-01700-x

**Published:** 2021-05-26

**Authors:** Ba’ Pham, Jelena Jovanovic, Ebrahim Bagheri, Jesmin Antony, Huda Ashoor, Tam T. Nguyen, Patricia Rios, Reid Robson, Sonia M. Thomas, Jennifer Watt, Sharon E. Straus, Andrea C. Tricco

**Affiliations:** 1 Knowledge Translation Program, Li Ka Shing Knowledge Institute, St. Michael’s Hospital, Unity Health Toronto, 209 Victoria St, Toronto, Ontario M5B 1T8 Canada; 2 Department of Software Engineering, University of Belgrade, Jove Ilica 154, Belgrade, 11000 Serbia; 3 Department of Electrical and Computer Engineering, Ryerson University, 350 Victoria Street, Toronto, Ontario M5B 2K3 Canada; 4 Epidemiology Division and Institute for Health Policy, Management, and Evaluation, Dalla Lana School of Public Health, University of Toronto, 155 College St Room 500, Toronto, Ontario M5T 3M7 Canada; 5 Queen’s Collaboration for Health Care Quality Joanna Briggs Institute Centre of Excellence, School of Nursing, Queen’s University, 99 University Ave, Kingston, Ontario K7L 3N6 Canada

**Keywords:** Systematic review, Scoping review, Text mining, Natural language processing, Machine learning, Classification model, Abstract screening, Automation

## Abstract

**Background:**

Current text mining tools supporting abstract screening in systematic reviews are not widely used, in part because they lack sensitivity and precision. We set out to develop an accessible, semi-automated “workflow” to conduct abstract screening for systematic reviews and other knowledge synthesis methods.

**Methods:**

We adopt widely recommended text-mining and machine-learning methods to (1) process title-abstracts into numerical training data; and (2) train a classification model to predict eligible abstracts. The predicted abstracts are screened by human reviewers for (“true”) eligibility, and the newly eligible abstracts are used to identify similar abstracts, using near-neighbor methods, which are also screened. These abstracts, as well as their eligibility results, are used to update the classification model, and the above steps are iterated until no new eligible abstracts are identified. The workflow was implemented in R and evaluated using a systematic review of insulin formulations for type-1 diabetes (14,314 abstracts) and a scoping review of knowledge-synthesis methods (17,200 abstracts). Workflow performance was evaluated against the *recommended practice* of screening abstracts by 2 reviewers, independently. Standard measures were examined: sensitivity (inclusion of all truly eligible abstracts), specificity (exclusion of all truly ineligible abstracts), precision (inclusion of all truly eligible abstracts among all abstracts screened as eligible), F1-score (harmonic average of sensitivity and precision), and accuracy (correctly predicted eligible or ineligible abstracts). Workload reduction was measured as the hours the workflow saved, given only a subset of abstracts needed human screening.

**Results:**

With respect to the systematic and scoping reviews respectively, the workflow attained 88%/89% sensitivity, 99%/99% specificity, 71%/72% precision, an F1-score of 79%/79%, 98%/97% accuracy, 63%/55% workload reduction, with 12%/11% fewer abstracts for full-text retrieval and screening, and 0%/1.5% missed studies in the completed reviews.

**Conclusion:**

The workflow was a sensitive, precise, and efficient alternative to the recommended practice of screening abstracts with 2 reviewers. All eligible studies were identified in the first case, while 6 studies (1.5%) were missed in the second that would likely not impact the review’s conclusions. We have described the workflow in language accessible to reviewers with limited exposure to natural language processing and machine learning, and have made the code available to reviewers.

**Supplementary Information:**

The online version contains supplementary material available at 10.1186/s13643-021-01700-x.

## Background

Well-conducted knowledge syntheses such as systematic reviews (SRs) provide valid evidence to inform decision-making [1]. However, SRs in healthcare can be time-consuming (e.g., 1 year), [2] labor-intensive (e.g., 1,139 person-hours, 5 reviewers) [3], and expensive (e.g., > $100,000) [4]. Automation that aims to minimize timelines, person-time, and cost expenditures is of interest to producers and users of knowledge synthesis internationally [5].

Study selection is one of the most important steps in the SR process [6]. The recommended practice is to have two reviewers screen titles and abstracts and to resolve discrepancies between reviewers in order to maximize the chance of identifying all eligible studies. Experienced reviewers are trained on eligibility criteria to reduce discrepancies and then conduct abstract screening independently [1, 6–8]. Abstract screening consumes about 25% of the total person-time per review, estimated to range between 1000 and 2000 person-hours [9]. Despite the rigorously recommended methods, sources of between-screener variation remain, including lack of information and varying interpretation for eligibility determination due to abstracts that report limited details on methods and results, and errors associated with distraction and fatigue, to name a few [10].

Recent advances in text mining (the science of extracting information from text) [11], and machine learning (the study of methods that learn patterns from data and make decisions with minimal human intervention) [12] have enabled solutions to previously intractable problems [13]. Since 2006, these advances have been adopted to support the automation of title and abstract screening [14]. Substantial progress has been made to partially automate the process [5], with tools deployed for real-world use [15–18], and their use described in review protocols [19]. With the increasing use of SR methods for different types of knowledge syntheses [20], continuing efforts have been expended to improve the performance of the automation tools [21, 22]. However, few tools for title and abstract screening attain the level of sensitivity and precision consistent with published benchmarks for pairs of human reviewers [1, 7, 8, 23].

We set out to develop a workflow *—* consecutive steps starting from importing titles and abstracts to a computing platform, to ending with a set of all eligible abstracts *—* for the automation of title and abstract screening that is comparable to the recommended practice of screening by two reviewers, independently. Our motivation was guided by making the workflow accessible to review teams conducting different types of knowledge syntheses, requiring minimal technical expertise and training. The current paper describes the design, development, and evaluation of the workflow using two case studies.

## Methods

### Workflow structure

The workflow is proposed to handle title and abstract screening for knowledge syntheses addressing clinical or non-clinical research questions. We adopted widely recommended text-mining and machine-learning methods to phase 1) process title-abstract texts into computing data; and phase 2) identify all eligible abstracts through repeated interactions between human reviewers and software, using a classification model and a nearest-neighbor search procedure. The two phases of the proposed workflow are outlined below, with terminologies related to text mining and machine learning described in a glossary (Additional File 1, Appendix A).

#### Phase 1: Preparation of abstracts for machine learning, and creation of the training dataset

**Fig. 1:**
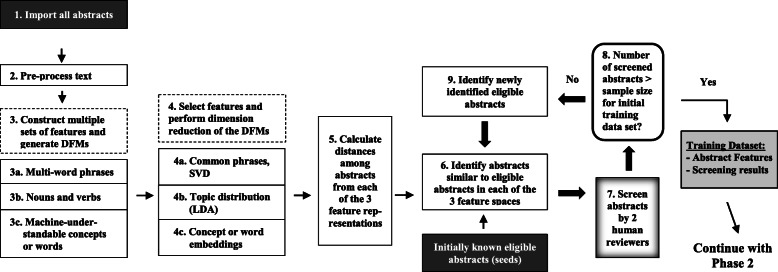
Workflow — Phase 1: Preparation of abstracts for machine learning, and creation of the training dataset. DFMs: document feature matrices. Dark lines denote iterations. Inputs to the workflow are denoted by black boxes. Box 4a. SVD: singular value decomposition. Box 4b. LDA: latent Dirichlet allocation for topic modeling. Box 4c. Concept or word embeddings are vector representations of words and their relationships. Outputs of this workflow phase are the abstract features, as well as the screening results of the abstracts reviewed by the 2 human reviewers in order to generate the training dataset, denoted by the gray box

In this phase, title-abstract texts (henceforth referred to as “abstracts”) are converted into numerical training data to train the machine learning model that classifies abstracts as eligible or ineligible. Using a simple example in Table 1, we illustrate the initial steps of transforming the content of a collection of abstracts into a “document-feature matrix,” where the rows denote the abstracts, the columns represent the features (e.g., in the form of words), and the values in the matrix are weights of the features in the corresponding abstracts. In this example, the weights are frequency of the feature in the abstract. Features with relatively high frequencies are selected and retained in a reduced document-feature matrix for further analyses. Figure 1 outlines the 9 steps of this phase.

**Table 1:**
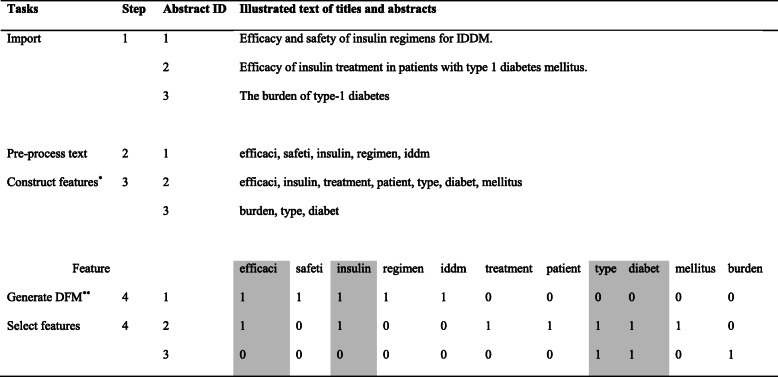
Illustrated workflow steps to process a simple collection of abstracts for machine learning classification

*DFM*  document-feature matrix. *IDDM* insulin-dependent diabetes mellitus. *The features in this example are words. ^**^The DFM contains frequency counts of the features. Highlighted columns in the DFM denote relatively high-frequency features that are selected and retained for further analysis

*Step 1. Import abstracts* — Titles and abstracts from the literature search are imported into a computing platform for text mining and machine learning, excluding citations with title only and no abstract.

*Step 2. Pre-process text* — Texts of each title and abstract are combined. Text is pre-processed through tokenization, lemmatization, parts of speech tagging, and as needed, semantic annotation. Tokenization is a step which splits longer strings of text into smaller pieces (tokens), such as words separated by white space. Lemmatization is a step which replaces a word by its normalized form (lemma). For example, “runs”, “ran”, and “running”, all have a common lemma, “run.” These steps ensure that words of the same meaning but expressed in slightly different forms are processed uniformly. Parts of speech tagging is done by marking up a word in a text as a noun, verb, pronoun, or adjective (among others), based upon the definition of the word and the context of its use (e.g., the context is defined by surrounding words).

Semantic annotation is a natural language processing task specifically designed for detecting and disambiguating terms mentioned in the texts into machine-understandable terminology, such as concepts in the Unified Medical Language System (UMLS) [24]. The UMLS Metathesaurus is a large biomedical thesaurus, organized by concept or meaning, and different expressions of the same concept are linked [25]. For example, “heart attack”, “coronary thrombosis”, and “cardiac arrest” are different expressions of the same concept — “Myocardial infarction” — registered in the UMLS Metathesaurus. To perform semantic annotation, we use RysannMD, a general-purpose biomedical semantic annotator that balances accuracy and computing time [24].

*Step 3. Construct 3 sets of features to represent the content of abstracts* — Once pre-processing of the abstracts is complete, the contents are summarized in a document-feature matrix where the columns represent each of 3 sets of features: (1) short phrases of 1, 2, or 3 contiguous words, (2) nouns and verbs, and (3) words for knowledge synthesis addressing the non-clinical review questions, or, in the case of clinical reviews, UMLS concepts. As we will show in the evaluation, multiple features are used to improve the sensitivity of the workflow, which aims to identify all eligible abstracts.

*Step 4. Select features and perform dimension reduction of the 3 document-feature matrices* — For the document-feature matrix with short phrases, only phrases common across abstracts are retained. A mathematical method known as Singular Value Decomposition (SVD) is then applied to the matrix to further reduce its dimension (e.g., from > 100,000 features to about 300 derived features) [26]. SVD results in a matrix transformation that obtains a more compact, computationally efficient representation of the abstracts, while at the same time preserving their semantic relations [27].

For the document-feature matrix of nouns and verbs, a topic-modeling method known as Latent Dirichlet Allocation is applied to identify common topics across abstracts [28, 29]. For, example, words like “cortisone”, “anti-viral”, and “rituximab”, if seen relatively frequently, might be grouped under the topic “Covid-19 treatments”, while “case”, “hospitalization”, “ventilator” might fall under “Covid-19 outcomes.” Only nouns and verbs are used since their use tends to generate consistent and meaningful topics [30]. The content of all abstracts is then summarized in a reduced document-feature matrix, with each row representing the probability distribution of the topics within an abstract (e.g., 300 common topics identified from all abstracts).

For the document-feature matrix of words for knowledge synthesis, only words with representations in GloVe are retained in the matrix. GloVe is an open-source project that has derived global vectors for word representations (commonly known as word embeddings) [31]. GloVe characterizes a word by other words that tend to appear with it, assuming that the words near a given word encode a large amount of information regarding that word’s meaning. Word embeddings model this contextual information by creating a lower-dimensional space such that words that appear in similar contexts are nearby in this new space (e.g., a 300-dimensional space). For example, semantically close words such as “effect” and “consequence” are mapped to close points in the low-dimensional space where representations of semantically unrelated words such as “effect” and “reject” are more distant. Each abstract is then replaced by a weighted average vector of the word embeddings that represent the words mentioned in the abstract, weighting on the frequency count of the words. A similar approach is applied to the document-feature matrix of UMLS concepts, with concept embeddings obtained from an open-source project that uses massive sources of multimodal medical data to derive concept embeddings [32].

*Step 5. Generate 3 distance matrices representing pairwise distances between the 3 numerical vectors that represent the abstracts* — Any two abstracts can be compared by taking the cosine of the angle between their vector representations (which are rows from a document feature matrix), with values approaching 1 denoting semantically close abstracts, and values approaching 0 denoting distant abstracts [27]. The distance between two abstracts is defined to be inversely proportional to the cosine of the angle [33, 34]. For the document-feature matrices derived with word embeddings, the word mover distance (WMD) between two abstracts represents the minimum total cosine distance that the embedded words of one abstract need to “travel” to reach the embedded words of the other abstract [35]. Compared to other distance measures, the use of WMD reduces error rates in document classification [35].

*Step 6. Identify abstracts similar to eligible seed abstracts* — This step requires a few seed abstracts (e.g., 3-5 abstracts) that are known to be eligible and are often identified in the preparation of the review protocol. For each of these seed abstracts, a fixed number, *k,* of nearest-neighbor abstracts are identified based upon the distances defined in *step 5*. For example, if we have 3 known eligible abstracts, with *k*=8 and three distance matrices, we would identify a batch of approximately 3 × 8 × 3 = 72 abstracts (with duplications removed). Information from a study examining characteristics of a representative sample of the population of SRs is used to guide the selection of *k* relative to the median number of 15 included studies (interquartile range: 8–25) [36].

*Step 7. Screen abstracts by pairs of reviewers* — The batch of abstracts identified in *step 6* is screened by two reviewers independently to identify eligible abstracts [1]. We expect a high proportion to be identified as eligible because their content is “near” those of known eligible seed abstracts. These screening results include results for both eligible and ineligible abstracts and will form part of the training dataset for the classification model (discussed below).

*Step 8. Assess the cumulative number of screened abstracts relative to a pre-set sample size for the training dataset* — If the cumulative number of screened abstracts falls below a pre-set sample size for the training dataset (e.g., 300–600 screened abstracts), additional eligible abstracts need to be identified.

*Step 9. Identify new eligible abstracts* — The screening results from *step 7* identified eligible abstracts in addition to the original seeds in *step 6*. These newly identified abstracts are then input to *step 6* as “seeds” so that yet additional and potentially eligible abstracts are screened for addition to the training dataset.

*Steps 6–9* are iteratively applied to the eligible abstracts newly identified in each iteration until the number of screened abstracts exceeds the pre-set sample size requirement for the training dataset. As we will show in the evaluation, phase 1 requires the screening of approximately 300 to 600 potentially eligible abstracts. We will also show that these abstracts are approximately 5 times more likely to be eligible than a randomly selected abstract from the literature search results.

#### Phase 2: Screening of abstracts through human-guided machine-learning

**Fig. 2:**
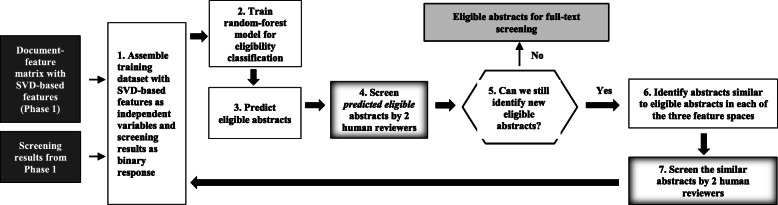
Workflow — Phase 2: Screening of abstracts through human-guided machine-learning. Dark lines denote iterations. Inputs to the workflow are denoted by black boxes. Outputs from the workflow are denoted by a gray box. Upper black box and box 1 — SVD, singular value decomposition

This phase aims to identify all abstracts that are eligible for full-text screening. It involves fitting a classification model to the training dataset, predicting eligible abstracts using the fitted model, screening the predicted eligible abstracts by 2 human reviewers to identify eligible abstracts, identifying abstracts similar to the eligible abstracts (using *step 6*), screening the similar abstracts by 2 human reviewers, updating the training dataset to include the screening results, updating the predictive model (which is then fit to the training dataset), and repeating the described steps until no newly eligible abstracts can be identified. Figure 2 outlines the 7 steps of this phase.

*Step 1. Assemble training dataset* — The screening results from *step 7, phase 1* are merged with the document-feature matrix with the SVD-based features (from *step 4, phase 1)* to generate a training dataset, with columns of the document-feature matrix being treated as predictors and the screening results as the binary response, for the development of the classification models [26, 27]. We will show in the evaluation that the classification model with SVD-based features attains a higher F1-score (the average of sensitivity and precision) than corresponding models based upon word-embedding and topic-modeling features (Additional File 1, Appendix B).

*Step 2. Train a random-forest model to classify eligible abstracts* — Among recently developed classification models for binary responses, a “random forest” is a combination of several decision trees (Glossary) [37] that attains relatively high precision, high sensitivity, and fast computing time [38]. A random-forest model is fit to the training dataset using the recommended method of multi-fold cross-validation to maximize the sensitivity of modeled prediction [39]. To deal with imbalanced distribution of eligible versus ineligible abstracts in each cross-validation fold (e.g., 5% versus 95%, respectively), the SMOTE algorithm is used to rebalance the distribution by generating synthetic abstracts with high eligibility probability (glossary, Additional File 1, Appendix A) [40].

*Step 3. Predict eligible abstracts* — The fitted model is used to predict eligible abstracts among all abstracts [39].

*Step 4. Screen predicted eligible abstracts* — The set of predicted eligible abstracts is screened by two human reviewers to identify eligible abstracts [1].

*Step 5. Assess for newly identified eligible abstracts in step 4* — The eligible abstracts identified in *step 4* are verified against the cumulative set of eligible abstracts identified so far to assess whether there are new eligible abstracts. If there are no new eligible abstracts, the process stops.

*Step 6. Identify abstracts similar to eligible abstracts* — For each of the newly identified abstracts in *step 4* and using the distance measures from *step 5 of phase 1*, a fixed number *k* of nearest-neighbor abstracts are identified as abstracts similar to the eligible abstracts (see also *step 6* of *phase 1*).

*Step 7. Screen the set of similar abstracts by pairs of reviewers* — The batch of similar abstracts identified in *step 6* is screened by 2 human reviewers, independently [1].

*Steps 1–7* are then iteratively applied, using an updated training set including the newly screened abstracts and their eligibility assessments, until no newly eligible abstracts can be identified in *step 5* (Fig. 2).

As we will show in the evaluation, phase 2 involves approximately 5 iterations, requires the screening of 37% to 45% of all abstracts, and identifies approximately 90% of the eligible abstracts among all abstracts screened by pairs of reviewers. We will show that compared to the reference standard of screening by pairs of reviewers, the workflow attains literature saturation, in the sense that additional eligible abstracts, if any and if identified by the reviewer pairs, would not change inferences in the knowledge synthesis.

Table A1 in Additional file 1 summarizes the implementation of the workflow in R (a programing language for statistical computing), using publicly available R packages for text mining and machine learning, notably the “caret” package that streamlines model training and evaluation [41]. Table A1 also displays values of key parameters in the workflow, including values for the main analysis and alternative values for sensitivity analysis. Values for the main analysis were selected through trial and error to optimize the performance of the workflow with respect to maximizing both the sensitivity of modeled classification and workload reduction (below). The selection necessarily involved uncertain judgment, and alternative parameter values likely to affect the workflow’s performance were identified for sensitivity analyses by varying the parameters one at a time.

Human and computing resources required for the implementation are reported in Additional File 1, Appendix A. The R code is reproduced in Additional File 1, Appendix C, and the case study original screening results can be replicated using the material available here (*https://knowledgetranslation.net/text-mining-to-support-abstract-screening-for-knowledge-syntheses-a-workflow-approach/*) [42, 43].

### Evaluating the performance of the workflow to identify eligible abstracts

From the database of our knowledge synthesis team, two case studies were selected based upon the following criteria: *i)* different types of knowledge synthesis, *ii)* availability of a review protocol, *iii)* broad eligibility criteria, and *iv)* the review results published in peer-reviewed journals. We selected a SR of the efficacy and safety of insulin formulations for patients with type-1-diabetes, and a scoping review on knowledge synthesis methods (Additional File 1, Appendix A) [42, 43]. The protocol and planned search strategies for each SR are accessible at *https://osf.io/xgfud*, and *https://bmcmedresmethodol.biomedcentral.com/articles*/*10.1186/1471-2288-12*-*114*, respectively. The search strategies may also be found within each result's publication [42, 43].

The screening results by the recommended practice of screening abstracts with 2 reviewers were considered the reference standard in the evaluation of the proposed workflow [1]. As such, for each of the two case studies, the results from the original review were used as the reference standard.

The proposed workflow was evaluated with respect to the following performance measures:

- ***N***_***P***_: the number of predicted eligible abstracts identified by the workflow at the end of phase 2,
- ***N***_***WF***_: the number of eligible abstracts (as determined by the reference standard) identified by the workflow at the end of phase 2,
- ***N***_***S***_: the number of eligible abstracts identified by human reviewers (the reference standard) after screening *all* abstracts (***N***),
- ΔN ***= N***_***S***_ − ***N***_***WF***_: The number of eligible abstracts missed by the workflow,
- The number of missed studies due to the full-text screening of the ***N***_***WF***_ eligible abstracts instead of full-text screening the ***N***_***S***_ eligible abstracts,
- Precision — the percentage of eligible abstracts predicted by the workflow that are confirmed via the reference standard (correctly predicted eligible abstracts) among all predicted eligible abstracts (***N***_***WF***_/***N***_***P***_) *100,
- Sensitivity — or recall, the percentage of correctly predicted eligible abstracts among the eligible abstracts identified via the reference standard (***N***_***WF***_/***N***_***S***_) *100,
- F1-score — the harmonic average of sensitivity and precision,
- Specificity — the percentage of correctly predicted ineligible abstracts among abstracts identified as ineligible via the reference standard ((***N*** − ***N***_***WF***_)/(***N*** − ***N***_***S***_)) *100,
- Accuracy — the percentage of correctly predicted (based on the reference standard) eligible or ineligible abstracts,
- Workload reduction — the difference between the total number of abstracts and the number of abstracts screened by the workflow, assuming each abstract is screened by two reviewers [11]
- Person-hours saved — the reduction in person-hours associated with the workload reduction, assuming that on average, a reviewer screens about 200 abstracts per hour [44, 45].

To provide benchmarking measures for the workflow’s performance, we conducted a literature review of studies reporting data on the performance of human reviewers conducting abstract screening for SRs (Additional File 1, Appendix A). Studies were identified from a recent SR of methods for study selection, including forward searching of citations of studies identified by the SR to identify eligible studies published after the SR [46].

## Results

**Fig. 3:**
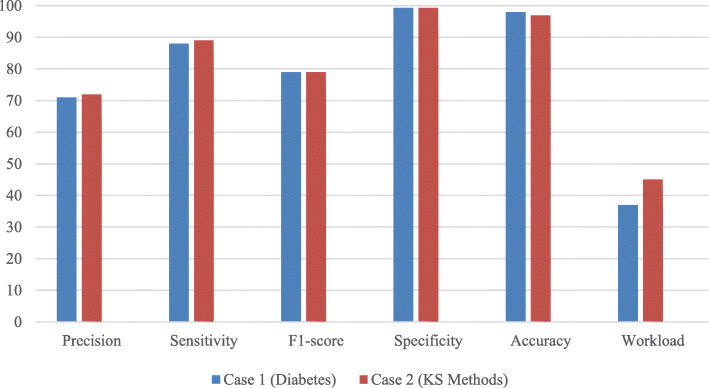
Workflow performance (%) vs recommended practice for abstracts screening (main analysis)

Table 2 displays the results of the evaluation of the workflow’s performance; step-specific results of the workflow performance are included in Additional File 1, Appendix B. Figure 3 displays the workflow’s performance on various performance measures relative to the recommended practice for abstracts screening. For the main analysis of the SR of type 1 diabetes, the workflow attained an 88% sensitivity, 71% precision, F1-score of 79%, 99.3% specificity, 98% accuracy, 63% workload reduction, or equivalently, a saving of 91 person-hours. Pairs of reviewers identified 743 eligible abstracts, while the workflow identified 655 eligible abstracts, or 88 fewer eligible abstracts. Compared to screening by pairs of reviewers, the workflow recommended 88 fewer eligible abstracts for full-text retrieval and screening, and this did not lead to any missed studies among the 80 studies included in the SR, which were identified via full-text screening of the 743 eligible abstracts.

**Table 2:** Evaluation of the workflow performance using the recommended practice as the reference standard

Case study	Type of analysis	Precision (%)	Sensitivity^*^ (%)	F1-score (%)	Specificity (%)	Accuracy (%)	N_WF_, N_S_, ΔN (# of eligible abstracts)	*# missed studies*^•^	Workload reduction^♦^ %	Hours^♣^ saved
SR — diabetes (14, 314 abstracts)	Main analysis^a^	71	88	79	99.3	98	655, 743, 88	0	63%	91 h
	SA: k-NN_2_ = 25	64	94	76	99.7	97	700, 743, 43	0	49%	70 h
	SA: *r* = 300	70	89	78	99.4	97	660, 743, 83	0	62%	89 h
	SA: k-NN_1_ = 15	68	89	77	99.4	97	664, 743, 79	0	61%	88 h
	SA: ϕ = 80%	72	88	79	99.3	98	653, 743, 90	0	63%	91 h
	SA: ϕ = 90%	68	88	76	99.3	97	653, 743, 90	0	64%	91 h
	SA: 2 distance measures^b^	77	84	80	99.1	98	623, 743, 120	0	74%	105 h
Scoping — KS methods (17, 200 abstracts)	Main analysis^a^	72	89	79	99.3	97	852, 957, 105	6	55%	95 h
	SA: k-NN_2_ = 25	65	95	77	99.7	97	907, 957, 50	3	39%	68 h
	SA: *r* = 300	72	90	80	99.4	97	858, 957, 99	5	54%	92 h
	SA: k-NN_1_ = 15	73	89	80	99.4	98	853, 957, 104	5	54%	94 h
	SA: ϕ = 80%	72	89	79	99.4	98	847, 957, 110	8	55%	95 h
	SA: ϕ = 90%	73	88	80	99.3	98	842, 957, 115	8	56%	96 h
	SA: 2 distance measures^b^	79	82	80	98.9	98	785, 957, 172	17	70%	119 h

*Sensitivity or recall; results of the sensitivity analyses are displayed in decreasing sensitivity of the workflow’s performance. ^**♣**^Person-hours that were tallied across reviewers. ^a^The main analysis was conducted with distance definitions from three feature representations (SVD-based, LDA-based and word-embedding features), a threshold ϕ = 70%, k-nearest-neighbor (k-NN_1_) for phase 1 of 8, k-NN for phase 2 (k-NN_2_) of 15, and initial sample size *r* = 600 (Table A1). SVD: singular value decomposition. *LDA* latent Dirichlet allocation. ^b^This sensitivity analysis used 2 distance measures from the SVD-based and word-embedding-based features. *SR* systematic review. *SS* scoping review. *KS* knowledge synthesis. *SA* sensitivity analysis. *NN* nearest-neighbors. ^♦^Workload reduction: the number of abstracts saved with the workflow, relative to the recommended practice of screening all abstracts by 2 reviewers. **N**_**WF**_—Number of eligible abstracts identified by the workflow. **N**_**S**_—Number of eligible abstracts identified via screening by 2 human reviewers (recommended practice). **Δ**_**N**_—The number of eligible studies missed by the workflow: **N**_**S**_ **− N**_**WF**_. ^•^The number of missed studies due to the full-text screening of the N_WF_ eligible abstracts instead of full-text screening the N_S_ eligible abstracts

For the main analysis of the scoping review of knowledge synthesis methods (Table 2), the workflow attained an 89% sensitivity, 72% precision, F1-score of 79%, 99.3% specificity, 97% accuracy, 55% workload reduction, or equivalently, a saving of 95 person-hours. Pairs of reviewers identified 957 eligible abstracts, while the workflow identified 852. Compared to screening by pairs of reviewers, the workflow recommended 105 fewer eligible abstracts for full-text retrieval and screening. This reduction led to 6 missed studies among the 409 studies included in the scoping review, which were identified via full-text screening of the 957 eligible abstracts (an error rate of 1.5%).

Table 2 also displays results of the sensitivity analysis. The workflow’s sensitivity increased to approximately 95% (from 88%/89%) with larger value *k* for the *k*-nearest-neighbors in phase 2 (*k* = 25, base value *k* = 15), but the workload reduction decreased by approximately 15%. Compared to the use of two feature representations in *step 3* of *phase 1* of the workflow, the use of the three feature representations led to higher sensitivity, which was reduced by 4%/7% by excluding the topic-modeling features. The results were robust against other parameter values, especially the threshold used to select common features in the derivation of the SVD-based features. The workflow performance was consistent regardless of the clinical and non-clinical topics, and across the SR and scoping review.

Given the computing resources (Additional File 1, Appendix A) and the implementation details of the workflow (Table A1, Additional File 1, Appendix B), it took approximately 6 computing hours to derive the document-feature matrix with SVD-based features, approximately 18 hours to derive the matrix with topic-modeling-based features, and approximately 60 hours to derive the matrix with word or concept embeddings. These initial steps to set up the workflow were time-consuming. Phase 1 required approximately 3 iterations, with no computing delays between iterations that were noticeable by reviewers. The saturation of newly identified eligible abstracts was attained after approximately 5 iterations of the steps in phase 2, with approximately 45-minute delays between iterations to update the classification model.

Results of the literature review evaluating the performance of human reviewers are given in Additional File 1, Appendix A. We identified three studies reporting benchmarking data, with varying review topics (postal survey methods in study 1 [45], diet research in study 2 [47], and brain injury in study 3 [48]), and varying reviewers’ experience (4 experienced reviewers, 12 reviewers with 6 experienced and 6 student reviewers, and 58 student reviewers, respectively). When reported, the sensitivity of reviewers ranged from 47% to 90% (the workflow’s sensitivity was 88–89% as reported above), specificity from 73% to 100% (workflow: >99%), precision from 55 to 90% (workflow: 71–72%), F1-score from 56 to 77% (workflow: 79%); and pairs of reviewers did not miss any eligible studies identified via full-text screening, with an estimated range of 0% to 1% of missed eligible studies (workflow: 0–1.5%).

## Discussion

Currently, the recommended practice is to screen titles and abstracts for knowledge syntheses with two reviewers, independently [1, 8, 23], and to err on the side of over-inclusion during screening [8]. Until now, the use of pairs of reviewers seemed to be the only known approach to reduce errors and subjectivity in study selection [6]. Compared to the recommended practice, the proposed workflow was sensitive, as it identified all eligible studies in the first case, while missing 6 studies (1.5%) in the second that would likely not impact the review’s conclusions. Thus, most importantly, we infer that in these cases, the results of the systematic reviews would not have changed had our semi-automated workflow originally been implemented.

The workflow was also reasonably precise, with approximately 7 truly eligible abstracts out of 10 predicted eligible. It was efficient, as it substantially reduced the workload of abstract screening by approximately 60%. Also, it referred 10% fewer abstracts for full-text retrieval and full-text screening, while ensuring literature saturation. Using two case studies, we showed that the workflow was generalizable to two different review topics and two different types of knowledge synthesis - a SR of a clinical review topic, and a scoping review of a methodology topic.

To overcome skepticism towards automation [5], both within the scientific community and among recognized SR, regulatory, and health technology assessment bodies, we designed the workflow with a strong emphasis on close interactions between human reviewers and the text-mining and machine-learning application. We endeavored to describe the proposed workflow in a way that is accessible to reviewers with limited exposure to text mining and machine learning, including a glossary of common terms (Additional File 1, Appendix A). We outlined the step-specific implementation in detail and implemented the workflow with publicly available software tools. Our study results can be replicated using materials available online, and the R codes are reproduced in Additional File 1, Appendix C. We hope all this would serve to facilitate the application, adoption and diffusion of the workflow into routine practice for review teams with interest in SR automation. We recommend our workflow be considered in a de novo implementation when the number of abstracts to be screened is at least 5000. As well, users may want to first consider piloting the workflow on a systematic review they completed, and comparing the results before using the workflow routinely.

We compared the workflow with other automation tools for abstract screening that are currently in use. Abstrackr, a commonly used tool, has been recently evaluated in four abstract-screening projects [49]. Across the projects, sensitivity ranged from 79 to 97%, and precision ranged from 15 to 65% [49]. The text-mining algorithm implemented in the online SR system EPPI-Reviewer has been evaluated recently using a case study [21]. According to the results, sensitivity could be very high (e.g., 99%) at reasonable precision (e.g., 50%). The text-mining algorithm implemented in the online SR system Rayyan was evaluated using a sample of 15 SRs [22]. According to the results, sensitivity ranged from 62 to 100%, and workload reduction ranged from 3 to 55%. The algorithm implemented in the Distiller SR platform was evaluated using a sample of 15 SRs, with workload reduction ranging from 9 to 62% [50]. In comparison, our proposed workflow was associated with high sensitivity (approaching 90%), high precision (approximately 70%), and high workload reduction (approximately 60%).

The workflow as described requires two systematic reviewers as part of the iterative human-machine-learning process. Certainly, one experienced reviewer could be considered instead, but we would expect inferior results. One reviewer could be used for the first few iterations, or for establishment of the training dataset, while the potential abstracts are “nearest” the seeds, and two reviewers could be used for the remaining iterations, without inferior results. This would be an item for further research.

There are limitations to our study. First, the evaluation of the workflow was retrospective, with potential bias associated with the fact that the reference standard was known prior to the evaluation. We only used two case studies in our evaluation because we wanted to provide a detailed analysis of the proposed automation approach. The step-specific methods we used in the workflow are simple; they work well together, but they might not be optimal for individual steps. The values we used for the parameters governing the steps of the workflow in Table A1 might not be the best values to optimize the overall performance of the workflow. In this regard, we did not try to establish optimal step-specific methods or parameter values, since we believe optimality would depend on the specific SRs or types of knowledge syntheses.

In the second case study (scoping review), the workflow missed 6 eligible studies that were identified by pairs of reviewers. We however believe that the workflow identified a saturated set of eligible studies, in the sense that the 6 missed studies would not change the inferences in the scoping review. These studies were eligible for inclusion for the scoping review but were not a major focus of the scoping review [51]. Another major limitation is that the workflow did not perform well with title-only citations, with low F-1 score (e.g., 7%, data not shown). Reviewers who are interested in using this workflow may have to apply the proposed automation approach to the set of titles only and no abstracts (separately from the handling of titles and abstracts) or manually screen citations with titles only.

## Conclusions

The workflow was a sensitive, precise and efficient alternative to the recommended practice of abstract screening with 2 reviewers, independently. All eligible studies were identified in the first case, while 6 studies (1.5%) were missed in the second that would likely not impact the review’s conclusions. We have described the workflow in language that is accessible to reviewers with limited exposure to natural language processing and machine learning as well as making the codes accessible to reviewers.

## Supplementary Information

**Additional File 1.** Supplementary Appendices

## Data Availability

The datasets used and the R codes implementing the workflow are available from a publicly accessible website (see “Methods”). The R codes are also reproduced in Additional File 1, Appendix C.

## Abbreviations

SR: Systematic review
DFMs: Document feature matrices
IDDM: Insulin-dependent diabetes mellitus
SVD: Singular value decomposition
LDA: Latent Dirichlet allocation
UMLS: Unified medical language system
WMD: Word mover distance
KS: Knowledge synthesis
SA: Sensitivity analysis
NN: Nearest neighbor
WLR: workload reduction
