# Supplementary material for: Text mining to support abstract screening for knowledge syntheses: a semi-automated workflow
Source: Syst Rev. 2021 May 26;10:156. doi: 10.1186/s13643-021-01700-x (PMC8152711; doi:10.1186/s13643-021-01700-x)
Supplement: Supplementary file 1 — Additional File 1. Supplementary Appendices [file 13643_2021_1700_MOESM1_ESM.docx]

Additional File 1: Supplementary Appendices

[Appendix A: Supplementary material for the Methods section of the main paper 3](#_Toc25058576)

[Human and computing resources required for implementing the workflow 3](#_Toc25058577)

[Case study # 1 3](#_Toc25058578)

[Case study #2: 3](#_Toc25058579)

[Literature review to derive benchmarks of performance by human reviewers 4](#_Toc25058580)

[Background 4](#_Toc25058581)

[Objectives 4](#_Toc25058582)

[Methods 4](#_Toc25058583)

[Results 4](#_Toc25058584)

[Summary 5](#_Toc25058585)

[Discussion 5](#_Toc25058586)

[Glossary of terms 6](#_Toc25058587)

[References 11](#_Toc25058588)

[Appendix B: Supplementary Tables 14](#_Toc25058589)

[Table B1. Implementation of the workflow 14](#_Toc25058590)

[Table B2. Performance of random-forest models with different document feature matrices 16](#_Toc25058591)

[Step-specific results of the workflow performance – Systematic review of type 1 diabetes 17](#_Toc25058592)

[Table B3. Base case analysis 17](#_Toc25058593)

[Table B4. Sensitivity analysis – Increasing k-nearest neighbor search in phase 2 18](#_Toc25058594)

[Table B5. Sensitivity analysis – Decreasing initial sample size 19](#_Toc25058595)

[Table B6. Sensitivity analysis – Increasing k-nearest neighbor search in phase 1 20](#_Toc25058596)

[Table B7. Sensitivity analysis – Decreasing threshold for selecting common features: 80% 21](#_Toc25058597)

[Table B8. Sensitivity analysis – Decreasing threshold for selecting common features: 90% 22](#_Toc25058598)

[Table B9. Sensitivity analysis – No distance matrix for topic-modeling-based features 23](#_Toc25058599)

[Step-specific results of the workflow performance – Scoping review of knowledge-synthesis methods 24](#_Toc25058600)

[Table B10. Base case analysis 24](#_Toc25058601)

[Table B11. Sensitivity analysis – Increasing k-nearest neighbor search in phase 2 25](#_Toc25058602)

[Table B12. Sensitivity analysis – Decreasing initial sample size 26](#_Toc25058603)

[Table B13. Sensitivity analysis – Increasing k-nearest neighbor search in phase 1 27](#_Toc25058604)

[Table B14. Sensitivity analysis – Decreasing threshold for selecting common features: 80% 28](#_Toc25058605)

[Table B15. Sensitivity analysis – Decreasing threshold for selecting common features: 90% 29](#_Toc25058606)

[Table B16. Sensitivity analysis – No distance matrix for topic-modeling-based features 30](#_Toc25058607)

[Appendix C: Workflow implementation in R codes 31](#_Toc25058608)

# Appendix A: Supplementary material for the Methods section of the main paper

## Human and computing resources required for implementing the workflow

This project was part of an on-going collaboration between a knowledge synthesis team, and a laboratory for systems, software and semantics.^1,2^ The project team is inter-disciplinary, including reviewers, review coordinators, review methodologists, data analysts and research scientists in semantic computing and knowledge engineering. All analyses were conducted in R.^3^ One author (JJ) initially developed the R codes for a workshop for doctoral students (all material available online).^4^ Another author (BP) adapted the R codes to the case studies, with problem solving support from other team members through 1-hour weekly meetings. Coding was initially done on a laptop (Intel core i3-4000M [CPU@2.4GHz](mailto:CPU@2.4GHz), 4GB RAM, 32-bit operating system) in R Studio,^5^ and run on a server (Linux Ubuntu, 64-bit operating system) through remote-communication freeware between the laptop and the server (Putty and Xming, see the references for the related URL’s),^6,7^ and parallel computing.^8^ The initial investment from the SR team was time and effort to foster collaboration with researchers with TM/ML expertise, to arrange for access to computing power, and to provide a supporting environment for SR automation.

Resources are required to integrate the implemented workflow into a review team, including access to TM/ML expertise and high performance computing (through a collaboration as described above), and the acquisition of TM skills by a team member. With TM tools that are increasingly accessible to non-specialists, the dedicated member could consider taking an introductory short-course in TM, or learning the relevant topics online. Our experience suggested that any reviewer with some interest in data analysis could learn to peruse the sample R codes to conduct the TM/ML analysis over a short time period (e.g., 3 months).

## Case study # 1

Comparative Efficacy and Safety of Intermediate-acting, Long-acting and Biosimilar insulins for Type 1 Diabetes Mellitus: A Systematic Review and Network Meta-Analysis. The review questions were Q1.What is the comparative clinical effectiveness and safety of intermediate-/long-acting insulin products and intermediate-/long-acting biosimilar insulin products in patients with T1DM? Q2.Should intermediate- and long-acting biosimilar insulin products be listed as a replacement for reference intermediate- and long-acting insulin (including insulin analogues) products when the latter products are not available (due to cost or supply issues)? The screening questions were formulated as follows: Q1. Does this study include adult patients (aged ≥16 years) with type 1 diabetes? Q2. Are patients treated with long-acting insulin, or intermediate-acting insulin, or biosimilar insulin preparations (24 formulations of insulin)? Q3. Does the study compare long-acting insulin, or intermediate-acting insulin, or biosimilar insulin preparations to one another, or Placebo, or no treatment? Q4. Is this an experimental, quasi-experimental, observational, or costing study?

The protocol and planned search strategy are accessible at https://osf.io/xgfud. The search strategies may also be found in the results publication: Tricco A. Comparative Efficacy and Safety of Intermediate-acting, Long acting and Biosimilar insulins for Type 1 Diabetes Mellitus: A Systematic Review and Network Meta-Analysis - A Study Protocol. Open Science Framework. 2017. https://osf.io/xgfud, Assessed 04 March 2021.

## Case study #2:

What is the most appropriate knowledge synthesis method to conduct a review? Protocol for a scoping review

A knowledge synthesis attempts to summarize all pertinent studies on a specific question, can improve the understanding of inconsistencies in diverse evidence, and can identify gaps in research evidence to define future research agendas. Knowledge synthesis activities in healthcare have largely focused on systematic reviews of interventions. However, a wider range of synthesis methods has emerged in the last decade addressing different types of questions (e.g., realist synthesis to explore mediating mechanisms and moderators of interventions). Many different knowledge synthesis methods exist in the literature across multiple disciplines, but locating these, particularly for qualitative research, present challenges. There is a need for a comprehensive manual for synthesis methods (quantitative/qualitative or mixed), outlining how these methods are related, and how to match the most appropriate knowledge synthesis method to answer a research question. The objectives of this scoping review are to: 1) conduct a systematic search of the literature for knowledge synthesis methods across multi-disciplinary fields; 2) compare and contrast the different knowledge synthesis methods; and, 3) map out the specific steps to conducting the knowledge syntheses to inform the development of a knowledge synthesis methods manual/tool.

Question 1: Is this a systematic review, according to the Cochrane Collaboration definition?

Question 2: Does this report describe the development, application, use or comparison of strategies or methods for synthesizing any type of evidence/literature. Only include novel knowledge synthesis methods.

Question 3: Is this report focused on a method related to health or philosophy?

The protocol and planned search strategy are accessible at https://bmcmedresmethodol.biomedcentral.com/articles/10.1186/1471-2288-12-114. The search strategies may also be found in the results publication: Tricco AC, Antony J, Soobiah C, Kastner M, Cogo E, MacDonald H, D'Souza J, Hui W, Straus SE. Knowledge synthesis methods for generating or refining theory: a scoping review reveals that little guidance is available. J Clin Epidemiol. 2016;73:36-42.

## Literature review to derive benchmarks of performance by human reviewers

### Background

Text mining classifiers are recommended as a second reviewer (with some reservation) according to a comprehensive systematic review of using text mining for study selection in systematic reviews.^9^ To our understanding, the performance of reviewers in abstract screening for study selection has not been empirically quantified.

### Objectives

To estimate the performance of human reviewers in order to provide benchmarking measures for evaluating machine-learning classifiers for abstract screening

### Methods

We conducted a meta-analysis of studies reporting reviewers’ performance. Studies were identified from three sources: 1) a recent systematic review of methods for study selection, data extraction and quality assessment;^10^ 2) a comprehensive systematic review of using text mining for study identification in systematic reviews;^9^ and 3) forward searching of citations of key studies identified in sources 1 and 2. Meta-analysis was conducted using the statistical package “mada” in R, as appropriate.^11^

### Results

The SR #1 included 9 studies evaluating methods for study selection, and three studies reported data on the performance of human reviewers.^12-14^ The SR #2 included 6 studies evaluating TM approaches as a second reviewer, and none of these studies reported data on reviewer’s performance. The forward search of key studies identified 3 potentially relevant citations; none of the three full-text reports included data on reviewer’s performance.

The study by Edwards et al. 1997 evaluated the performance of 4 reviewers who were experienced with systematic reviews.^12^ The review topic was related to strategies to influence response to postal survey. The literature search yielded 22,571 abstracts. The study was designed so that each pair of reviewers screened 11,286 abstracts, such that each abstract was seen by 2 reviewers. Abstract screening by the 4 reviewers identified 301 potentially relevant citations. Of these, the authors were able to retrieve 273 complete reports (they could not find full-text reports of the remaining 28 potentially relevant citations). In total, 156 abstracts (156/273, or 57%) met the inclusion criteria. Reviewer 1 identified 79 eligible reports, reviewer 2 identified 60 eligible reports, reviewer 3 identified 67 eligible reports and reviewer 4 identified 81 eligible reports. Across reviewers, the sensitivity estimate was 0.57 (95% confidence interval: 0.48, 0.66), specificity was 0.994 (95% CI: 0.992, 1.0), precision 0.77 (range: 0.55, 0.90) and F1-score 0.65 (range 0.56, 0.77).

According to results of the Edwards et al. study, single reviewers missed on average 8 per cent of eligible reports (range 0 to 24 per cent), whereas pairs of reviewers did not miss any (range 0 to 1 per cent).

The study by Cooper et al. 2006 included 12 reviewers, 6 experienced reviewers and 6 student reviewers.^14^ The review topic was related to diet research. The literature search yielded 90 abstracts for screening. The study reported sensitivity and specificity estimates for each reviewer, with no differences between experienced reviewers and student reviewers. The average sensitivity was 0.84 (range 0.72, 0.90) and specificity 0.85 (0.73, 0.95). Precision and F1-score could not be estimated with the reported data. Meta-analysis was used to estimate the relationship between sensitivity and specificity. If one wished to set sensitivity as high as 0.95, the corresponding specificity could have dropped to 0.50.

The study by Ng et al. 2014 included 58 student reviewers.^13^ The review topic was related to hypothermia for traumatic brain injury. The literature search yielded 650 abstracts for screening. Sensitivity ranged from 0.47 to 0.67, specificity ranged from 0.93 to 0.97. No other performance statistics were possible with the reported data.

### Summary

Overall, we identified three studies reporting data on the benchmarking performance measures, with varying review topics (postal survey methods in study 1,^12^ diet research in study 2,^14^ and brain injury in study 3)^13^ and reviewers (4 experienced reviewers, 12 reviewers with 6 experienced and 6 student reviewers, and 58 student reviewers, respectively). When reported, the sensitivity of reviewers when screening abstracts ranged from 47% to 90%, specificity from 73% to 100%, precision from 55% to 90%, F1-score from 56% to 77%; and pairs of reviewers did not miss any eligible studies, ranging from 0% to 1%.

### Discussion

- Very limited data
- Expected false negative rate of 0% to 1% with pair of reviewers, assessing abstracts independently.
- If one wishes to set sensitivity of ≥0.95, the expected specificity would be around 0.50.
- The results support a benchmark F1-score of 0.65, with plausible values ranging from 0.56 to 0.77.

## Glossary of terms

This glossary was compiled mainly using material from the Encyclopedia for machine learning and data mining, version 2, 2017.^15^ Otherwise, specific sources are cited as part of the description of items.

*Corpus*: a large and structured set of texts usually electronically stored and processed.

*Text mining:* the science of extracting information from text.

*Machine learning:* the study of algorithms and mathematical models that can learn patterns from data and make decisions with minimal human intervention.

*Workflow:* the sequence of steps involved in moving from the beginning to the end of a working process.^16^

*Natural language processing:* the science of how to program computers to process and analyze large amounts of natural language data.

*Text cleaning:* the process of detecting and correcting (or removing) corrupt or inaccurate records from the data. For the workflow, we removed all text related to copyright information from the imported citations.

*Pre-process text:* when text is represented as a sequence of characters, the usual step is to convert it into a sequence of words. A word is thought of as a sequence of alphabetic characters delimited by whitespace and/or punctuation. For the workflow, the distinction between uppercase and lowercase is largely irrelevant; hence, all texts are converted into lowercase. Other pre-processing steps include text cleaning; removing numbers, punctuation marks, symbols, and so on; stemming; lemmatization; removing words with 1 or 2 characters; and removing stop words that contribute little to the understanding of the content (e.g., “and”, “the”, “a”). Pre-processing is done so that words of the same meaning but in slightly different forms can be processed consistently across the corpus.

*Tokenization:* Tokenization is a step which splits longer strings of text into smaller pieces, or tokens.^17^ Larger chunks of text can be tokenized into sentences; sentences can be tokenized into words, etc. Further processing is generally performed after a piece of text has been appropriately tokenized. Tokenization is also referred to as text segmentation or lexical analysis. Sometimes segmentation is used to refer to the breakdown of a large chunk of text into pieces larger than words (e.g. paragraphs or sentences), while tokenization is reserved for the breakdown process which results exclusively in words.

*Normalization:* Before further processing, text needs to be normalized.^17^ Normalization generally refers to a series of related tasks meant to put all text on a level playing field: converting all text to the same case (upper or lower), removing punctuation, converting numbers to their word equivalents, and so on. Normalization puts all words on equal footing, and allows processing to proceed uniformly.

*Stemming:* each word is replaced by its stem where the ending of the word is typically chopped off (e.g., walking => walk).

*Lemmatization:* replace a word by its normalized form (lemma), to reduce many different forms of the same word, such as those related to plural, gender influence on verb form, and so on. For example, the words “runs”, “ran”, “running” are all forms of the lemma “run”.

*Part-of-speech tagging:* In corpus linguistics, part-of-speech tagging (POS tagging or PoS tagging or POST), also called grammatical tagging or word-category disambiguation, is the process of marking up a word in a text (corpus) as corresponding to a particular part of speech, based on both its definition and its context—i.e., its relationship with adjacent and related words in a phrase, sentence, or paragraph. POS tagging could be also defined as a process in which each word in a text is assigned its appropriate morphosyntactic category (for example noun-singular, verb-past, adjective, pronoun-personal, and the like). It therefore provides information about both morphology (structure of words) and syntax (structure of sentences). This disambiguation process is determined both by constraints from the lexicon (what are the possible categories for a word?) and by constraints from the context in which the word occurs (which of the possible categories is the right one in this context?).

*Dependency parsing:* A dependency parser analyzes the grammatical structure of a sentence, establishing relationships between "head" words and words, which modify those heads. Dependency parsing is the task of extracting a dependency parse of a sentence that represents its grammatical structure and defines the relationships between “head” words and words, which modify those heads.

*Semantic annotation:* To enable effective and efficient use of the available and continuously evolving textual information repositories, the biomedical community has long been experimenting with and making use of Natural Language Processing (NLP) methods and techniques. One focus is on semantic annotation, an NLP task specifically designed for detecting and disambiguating biomedical concepts mentioned in the text. For instance, in the sentence ‘‘Heart attack victims, sometimes caused by MS, often suffer brain damage”, the role of a semantic annotation process would be to automatically recognize that the term ‘‘Heart attack” refers to the concept Myocardial Infarction (C0027051) from the UMLS Metathesaurus and hence establish a connection between that concept and its textual representation in the given sentence. Likewise, the term ‘‘brain damage” would be connected to the concept Brain Injuries (C0270611). By connecting terms with machine understandable concepts, semantic annotation is setting the grounds for (full or partial) automation of various laborious and time-consuming tasks such as search, classification, and/or organization of biomedical resources.

*Feature construction:* Feature construction in text mining consists of various techniques and approaches which convert textual data into a feature-based representation.

*Feature vector representation*: In the feature vector representation of a citation *d*, the feature corresponding to the word *w* would tell something about the presence of the word *w* in this citation: either the frequency (number of occurrences) of *w* in *d*, or a simple binary value (1 if present, 0 if absent), or it can further be modified by the term frequency – inverse document frequency (TF-IDF) weighting.

*Bag-of-words model:* a simple natural language processing model in which the content of a document is reduced to words that capture its content, ignoring the order but maintaining the multiplicity of words.

*Vector space model:* Document classification is usually performed by representing documents as vectors of features; usually the features are words so each document is represented by a vector of words in the corpus that includes all records. This representation is referred to as the “vector space model” representation.

*Term frequency:* the number of occurrence of a term in the document.

*Document frequency:* the number of times the term appears in the corpus.

*TF-IDF weighting:* Features (or terms) are weighted proportional to the term frequency (the number of occurrence of a term in the document), but offset by the document frequency (the number of times the term appears in the corpus), since terms common across documents provide little information useful for the classification. The term weight is proportional to the term frequency and inversely proportional to the document frequency.^18^

*n-gram:* Multi-word phrases sometimes have a meaning that is not adequately covered by the individual words of the phrase (e.g., technical terms). An n-gram is a multi-word feature, defined as a sequence of *n* adjacent words from the document (e.g., *n*=2 to 5). Many of the n-grams are incidental and irrelevant, but some of them may be valuable and informative phrases in the classification application. After pre-processing of the input text, single words, 2-grams and 3-grams were used as features in the workflow. The use of n-grams as features has been found to be beneficial, e.g., for the classification of very short documents.^19^

*Feature representation (feature weight)*: Each unique feature in the corpus is given a weight that is proportional to the number of occurrences of the feature in a citation (i.e., the term frequency), but inversely proportional to the number of times the feature appears in the corpus document frequency (i.e., the document frequency), since features common across citations provide little information useful for the classification.^18^

*Feature selection:* The process of selecting a subset of features used to represent the data. For the workflow, this was done by keeping the most relevant features using a cut-off based on the distribution of the feature weights (e.g., 90th, 95th percentiles).

*Document-feature matrix (document-term matrix)*: a mathematical matrix used to represent the citations in the corpus by the rows, the features identified in the corpus by columns, and the relevance of the features in the classification of citations by the feature weights.

*Feature projection techniques:* Techniques to find new data axes that retain the data structure and preserve its variance as closely as possible. For the workflow, we used singular value decomposition as the feature projection technique.

*Singular value decomposition (SVD)*: This linear algebra method for feature selection is also called latent semantic indexing (LSI) in text mining. LSI uses SVD to express the document-feature *d* x *n* matrix *X* as the product of three matrices, *T * S * D*, where *T* is a *d* x *r* orthonormal matrix, D is a *r* x *n* orthonormal matrix, and S is a *r* x *r* diagonal matrix containing the singular values of *X*. Here, *r* denotes the rank of the original matrix *X*. Let *T^(m)^* be the matrix consisting of the left *m* columns of *T*, let *D^(m)^* be the matrix consisting of the top *m* rows of *D*, and let *S^(m)^* be the top left *m* x *m* sub-matrix of *S*. Then it turns out that *X^(m)^* = *T^(m)^* ** S^(m)^* ** D^(m)^* is the best rank-*m* approximation of the original *X* (best in the sense of minimizing the norm of *X – X^(m)^*). Thus, the *i*-th column of *D^(m)^* can be seen as a vector of *m* new features representing the *i*-th document of the original matrix *X*, and the product *T^(m)^* ** S^(m)^* can be seen as a set of *m* new coordinate axes. The new feature vectors (columns of *D^(m)^*) can be used instead of the original feature vectors in *X*.^20^

*Topic modeling:* Topic modeling is a machine learning technique for discovering semantic topics from a document collection. It typically assumes that a document is a multinomial distribution over latent topics, and a topic is a multinomial distribution over words. By capturing the co-occurrence statistics of words in the documents, it uncovers these distributions, which indicate important semantic relationships.

*Word embeddings:* A word can be characterized by "the company it keeps", thus context words that appear around a given word encode a large amount of information regarding that word’s meaning. Word embeddings model this contextual information by creating a lower-dimensional space such that words that appear in similar contexts will be nearby in this new space.^21^

*Word mover distance:* The distance measures the dissimilarity between two text documents as the minimum amount of distance that the embedded words of one document need to “travel” to reach the embedded words of another document.^22^

*Random Forest (RF):* RF is a collection of decision trees (DT).^23^ For example, the DT algorithm repeatedly splits the *v*-dimensional space described in *step 4* of the workflow, and the sub-regions of the space resulting from the initial split are further split until say for example each sub-region is with >5 observations; each split is made based on the variable in which the split maximizes the separation of eligible citations from ineligible ones. A DT model tends to be sensitive to small changes in the training data used in the development of the model. Also, a DT model tends to involve multiple splits to fit to the specificity of the training data, resulting in problems with generalizing the classification results to new data.

The RF algorithm includes two maneuvers to alleviate these issues. First, it grows DTs on bootstrapped training sets. The bootstrapped sets are obtained by repeatedly sampling citations from the original training dataset, with replacement. When building these trees, each time a split in a tree is considered, only a random sample of the variables (features) is chosen as split candidates. By growing the collection of trees this way, the trees are independent. Using vote counting across the classifications of the trees, the RF algorithm improves generalization of the classification and reduces the variation in the prediction.^23^

*Support Vector Machine (SVM)***:** The SVM algorithm performs classification in a multidimensional space by finding a hyperplane that best separates eligible from ineligible citations in the *v*-dimension space (*step 4*).^24^ In geometry, a hyperplane is a subspace whose dimension is one less than that of its ambient space (e.g., if the ambient space is a 2-dimensional plane, then its hyperplanes are the 1-dimensional lines). Support vectors are data points nearest to the hyperplane, the critical points that, if removed, would alter the position of the separating hyperplane. The algorithm allows soft margin that cushions the hyperplane to allow for errors to be made while fitting the SVM model, with the term “softness” referring here to setting the cost of misclassification low when the SVM is undergone training. By allowing errors in the training, the algorithm produces a more generalizable classification model. The adopted algorithm included a kernel function to project the input space of *v* dimensions into a higher-dimension space where eligible and ineligible citations could be linearly separated through a hyperplane.^25^

***k****-fold cross-validation:* The random forest and support vector machine models were fitted to the train dataset using the method of *k-fold* cross-validation (e.g., *10-fold*).^23^ Each model was optimized, through cross-validation, by searching a grid of plausible values of model parameters for values that would maximize the model performance, with respect to the area under the receiver-operating curve.^26^ The receiver-operating curve was used to evaluate the model predictions versus the reference standard from human reviewers. The adopted *k-fold* cross-validation method consisted of randomly splitting the train dataset into 10 folds and training each model in 10 cycles, such that in each cycle, training was conducted with 9 folds, and model performance was evaluated on the remaining 1 fold. Finally, model performance was averaged across the 10 cycles, thus reducing prediction variation and improving the validity of the predicted classification.

*Imbalanced distribution:* A dataset is imbalanced if the classification categories are not approximately equally represented. In the workflow, we dealt with imbalanced distribution of eligible vs. ineligible citations in the cross-validation folds by using the SMOTE algorithm - a resampling scheme that effectively rebalances the distribution using synthetic citations with high probability of being eligible.^27^

*The SMOTE algorithm* – Abstract of the cited article:^27^ A dataset is imbalanced if the classiﬁcation categories are not approximately equally represented. Often real-world data sets are predominately composed of “normal” examples with only a small percentage of “abnormal” or “interesting” examples. It is also the case that the cost of misclassifying an abnormal (interesting) example as a normal example is often much higher than the cost of the reverse error. Under-sampling of the majority (normal) class has been proposed as a good means of increasing the sensitivity of a classiﬁer to the minority class. This paper shows that a combination of our method of over-sampling the minority (abnormal) class and under-sampling the majority (normal) class can achieve better classiﬁer performance (in ROC space) than only under-sampling the majority class. This paper also shows that a combination of our method of over-sampling the minority class and under-sampling the majority class can achieve better classiﬁer performance (in ROC space) than varying the loss ratios in Ripper or class priors in Naive Bayes. Our method of over-sampling the minority class involves creating synthetic minority class examples. Experiments are performed using C4.5, Ripper and a Naive Bayes classiﬁer. The method is evaluated using the area under the Receiver Operating Characteristic curve (AUC) and the ROC convex hull strategy.

## References

1. KT. Knowledge Translation Program. <https://knowledgetranslation.net/>.

2. LS3. Laboratory for Systems, Software and Semantics. <http://ls3.rnet.ryerson.ca/>.

3. Team RC. R: A language and environment for statistical computing. 2013; <http://www.R-project.org/>.

4. Jovanović J. Society for Learning Analytics Research. 2018; <https://solaresearch.org/events/lasi/lasi-2018/lasi18-workshops/>.

5. RStudio. R Studio.

6. Putty. <https://www.putty.org/>.

7. Xming. <https://sourceforge.net/projects/xming/>.

8. Zhou M. High performance computing in R using doSNOW package 2014.

9. O'Mara-Eves A, Thomas J, McNaught J, Miwa M, Ananiadou S. Using text mining for study identification in systematic reviews: A systematic review of current approaches. *Systematic Reviews.* 2015;4(1).

10. Robson RC, Pham B, Hwee J, et al. Few studies exist examining methods for selecting studies, abstracting data, and appraising quality in a systematic review. *J Clin Epidemiol.* 2018.

11. Doebler P, Holling H. Analysis of Diagnostic Accuracy with mada 2017; <https://cran.r-project.org/web/packages/mada/vignettes/mada.pdf>.

12. Edwards P, Clarke M, DiGuiseppi C, Pratap S, Roberts I, Wentz R. Identification of randomized controlled trials in systematic reviews: accuracy and reliability of screening records. *Stat Med.* 2002;21(11):1635-1640.

13. Ng L, Pitt V, Huckvale K, et al. Title and Abstract Screening and Evaluation in Systematic Reviews (TASER): a pilot randomised controlled trial of title and abstract screening by medical students. *Syst Rev.* 2014;3:121.

14. Cooper M, Ungar W, Zlotkin S. An assessment of inter-rater agreement of the literature filtering process in the development of evidence-based dietary guidelines. *Public Health Nutr.* 2006;9(4):494-500.

15. *Encyclopedia of Machine Learning and Data Mining.* New York: Springer 2017.

16. Van der Aalst WMP, Ter Hofstede AHM, Kiepuszewski B, Barros AP. Workflow patterns. *Distributed and Parallel Databases.* 2003;14(1):5-51.

17. Nuggets K. A General Approach to Preprocessing Text Data. 2019; <https://www.kdnuggets.com/2017/12/general-approach-preprocessing-text-data.html>. Accessed Feb. 10, 2019, 2019.

18. Robertson S. Understanding inverse document frequency: On theoretical arguments for IDF. *Journal of Documentation.* 2004;60(5):503-520.

19. Mladenic D, Grobelnik M. Feature selection on hierarchy of web documents. *Decision Support Systems.* 2003;35(1):45-87.

20. Brank J, Mladeni D, Grobelnik M. *Feature Construction in Text Mining.* New York: Springer 2017.

21. Andrew L. Beam, Benjamin Kompa, Inbar Fried, et al. Clinical Concept Embeddings Learned from Massive Sources of Multimodal Medical Data. 2018.

22. Kusner M, Sun Y, Kolkin N, Weinberger K. From Word Embeddings To Document Distances. *Proceedings of the 32 nd International Conference on Machine Learning, Lille, France, 2015.*

23. Gareth James, Daniela Witten, Hastie T, Tibshirani R. *An Introduction to Statistical Learning with Applications in R* New York: Springer Science, Business Media 2017.

24. Cortes C, Vapnik V. Support-vector network. *Machine Learning.* 1995;20 (1):25.

25. Meyer D. Support Vector Machines. 2017; <https://cran.r-project.org/web/packages/e1071/vignettes/svmdoc.pdf>.

26. Cohen AM, Smalheiser NR, McDonagh MS, et al. Automated confidence ranked classification of randomized controlled trial articles: An aid to evidence-based medicine. *Journal of the American Medical Informatics Association.* 2015;22(3):707-717.

27. Chawla NV, Bowyer KW, Hall LO, Kegelmeyer WP. SMOTE: Synthetic minority over-sampling technique. *Journal of Artificial Intelligence Research.* 2002;16:321-357.

28. *Statistical sofware R version 3.3.0 (2016-05-03)* [computer program]. 2016.

29. Tricco A. *Comparative Efficacy and Safety of Intermediate-acting, Long acting and Biosimilar insulins for Type 1 Diabetes Mellitus: A Systematic Review and Network Meta-Analysis - A Study Protocol.* Open Science Framework;2017.

30. Kastner M, Tricco AC, Soobiah C, et al. What is the most appropriate knowledge synthesis method to conduct a review? Protocol for a scoping review. *BMC Med Res Methodol.* 2012;12:114.

31. Kendall Fortney. Pre-Processing in Natural Language Machine Learning. 2017; <https://towardsdatascience.com/pre-processing-in-natural-language-machine-learning-898a84b8bd47>.

32. Quanteda. Quantitative analysis of textual data. <https://quanteda.io/>.

33. Institute of Formal and Applied Linguistics. UDPipe. 2019; <http://ufal.mff.cuni.cz/udpipe#introduction>.

34. Cuzzola J, Jovanović J, Bagheri E. RysannMD: A biomedical semantic annotator balancing speed and accuracy. *Journal of Biomedical Informatics.* 2017;71:91-109.

35. Pennington J, Socher R, Manning C. GloVe: Global Vectors for Word Representation. 2014.

36. Crone SF, Lessmann S, Stahlbock R. The impact of preprocessing on data mining: An evaluation of classifier sensitivity in direct marketing. *European Journal of Operational Research.* 2006;173(3):781-800.

37. Manning C, Prabhakar Raghavan, Schutze. H. *Introduction to information retrieval.* Cambridge, UK: Cambridge University Press; 2008.

38. Deerwester S, Dumais ST, Furnas GW, Landauer TK, Harshman R. Indexing by latent semantic analysis. *Journal of the American Society for Information Science.* 1990;41(6):391-407.

39. Irlba. Fast Truncated Singular Value Decomposition and Principal Components Analysis for Large Dense and Sparse Matrices. 2018; <https://cran.r-project.org/web/packages/irlba/irlba.pdf>.

40. Nadkarni PM, Ohno-Machado L, Chapman WW. Natural language processing: an introduction. *Journal of the American Medical Informatics Association.* 2011;18(5):544-551.

41. Blei DM. Probabilistic topic models. *Communications of the ACM.* 2012;55(4):77-84.

42. Blei DM, Ng AY, Jordan MI. Latent Dirichlet allocation. *Journal of Machine Learning Research.* 2003;3(4-5):993-1022.

43. Grün B, K H. topicmodels: An R Package for Fitting Topic Models. . *J Stat Softw.* 2011;40(3):1-30.

44. R Core Team. R: A Language and Environment for Statistical Computing. 2018; <https://www.R-project.org/>, August 2019.

45. Cuzzola J, Jovanovic J, E B. RysannMD: (RY)erson (S)emantic (ANN)otator 2017; <http://denote.rnet.ryerson.ca/RysannMD/>.

46. Foltz PW, Kintsch W, Landauer TK. The measurement of textual coherence with latent semantic analysis. *Discl Process.* 1998;25(2-3):285-307.

47. Page MJ, Shamseer L, Altman DG, et al. Epidemiology and Reporting Characteristics of Systematic Reviews of Biomedical Research: A Cross-Sectional Study. *PLoS Med.* 2016;13(5):e1002028.

48. Higgins J, Green S. *Cochrane Handbook for Systematic Reviews of Interventions Version 5.1.0.* The Cochrane Collaboration; 2011.

49. Landauer TK, Foltz PW, Laham D. An introduction to latent semantic analysis. *Discl Process.* 1998;25(2-3):259-284.

50. Kuhn M. Building Predictive Models in R Using the caret Package. 2008.

51. Bradley AP. The use of the area under the roc curve in the evaluation of machine learning algorithms. *Pattern Recognition.* 1997;30(7):1145-1159.

52. DMwR. Functions and data for ``Data Mining with R''. 2013; <https://cran.r-project.org/web/packages/DMwR/DMwR.pdf>.

# Appendix B: Supplementary Tables

## Table B1. Implementation of the workflow

| Step | Details of the implementation or evaluation | Value | Ref. |
| --- | --- | --- | --- |
| *Phase 1 – Figure 1 in the main article* | |  |  |
| 1. *Import* *of citations* | |  | ^28^ |
|  | Systematic review of insulin formulation for patients with type 1 diabetes |  | ^29^ |
|  | # eligible abstracts / total # of abstracts (%) at the abstract screening level | 743 / 14,314 (3%) |  |
|  | # Initially known eligible abstracts to start the search for abstracts similar to eligible abstracts | 5 |  |
|  | # studies included in the completed systematic review | 80 |  |
|  | Scoping review of knowledge synthesis methods |  | ^30^ |
|  | # eligible abstracts / total # of abstracts (%) at the abstract screening level | 953 / 17,200 (6%) |  |
|  | # Initially known eligible abstracts to start the search | 4 |  |
|  | # studies included in the full-text screening (the scoping review is on-going) | 409 |  |
| 1. *Pre-processed text* | |  |  |
|  | Managing and preparing text for text-mining and machine-learning analyses | R package “quanteda” | ^31^ ^32,33^ |
|  | Part-of-speech tagging | R package “UDpipe” | ^33^ |
|  | Semantic annotation of abstracts of clinical studies using the biomedical semantic annotator RysannMD | RysannMD | ^34^ |
|  | Matching words in abstracts of non-clinical studies to words in pre-trained word vectors  Pre-trained word vectors with 840 billion tokens, 2.2 million vocabulary, 300-dimension vectors | Glove | ^35^ |
| 1. *Construct multiple sets of features* | |  | ^36^ ^32^ |
|  | Generate document-feature matrix (DFM) with short phrases of 1, 2, or 3 contiguous words | 1-, 2-, 3-grams | ^31^ ^32^ |
|  | Generate DFM with nouns and verbs | Nouns and verbs | ^31^ ^33^ |
|  | Generate DFM matrix with UMLS clinical unique identifiers (CUIs) for abstracts of clinical studies | RysannMD, CUIs | ^21,34^ |
|  | Generate DFM with words matching words in GloVe pre-trained word vectors for abstracts of non-clinical studies | Glove pre-trained words | ^35^ |
| 1. *Select features and perform dimension reduction on three document-feature matrices (DFM)* | |  | ^37^ ^32^ |
|  | *I.* DFM with short phrases | 1-, 2-, 3-grams | ^31^ ^32^ |
|  | Select features using a threshold based on the distribution of the feature weights in the DFM |  |  |
|  | Use a threshold based upon the percentile φ of the distribution | 70% (80%, 90%) |  |
|  | Use singular value decomposition (SVD) to reduce the DFM to 300-feature dimension | 300 features | ^38^ ^39^ |
|  | *II.* DFM with nouns and verbs | Nouns and verbs | ^40^ ^33^ |
|  | Use topic modeling with Latent Dirichlet Allocation to reduce the DFM to a DFM with 300 topics as features | 300 topics | ^41,42^ ^43^ |
|  | *III.* DFM with Glove words as features for abstracts of non-clinical studies | Pre-trained words | ^35^ |
|  | Derive abstract representation as the weighted average of word vectors of 300 dimensions, weighting on the frequency of words in the abstract | Abstract representation | ^44^ |
|  | *III.* DFM with clinical unique identifiers CUIs as features for abstracts of clinical studies | RysannMD, CUIs | ^21,34^ |
|  | Retain CUIs with ≥ a threshold of accuracy of the term-to-concept mapping | ≥50% | ^21,34^ |
|  | Derive abstract representation as the weighted average of word vectors of 500 dimensions, weighting on the frequency of words in the abstract | 500 dimensions | ^45^ |
| 1. *Calculate distances among abstracts using distance matrices corresponding to three DFMs (step 4)* | |  |  |
|  | Calculate cosine distance between the angle of the feature-representation of two abstracts | Cosine distance | ^46^ |
|  | Calculate word mover distance (WMD) between the weighted averages of the word embeddings of 2 abstracts | WMD, cosine distance | ^22^ |
| 1. *Identify abstracts similar to eligible abstracts in each of the three feature-based representations* | |  |  |
|  | Select the *k* nearest-neighbors (k-NN) of an eligible abstract to be included in the train dataset^♣^ | 8[15]* | ^47^ |
| 1. *Screen abstracts by pairs of reviewers, independently* | | 2 reviewers | ^48^ |
| *8. Assess the number of screened abstracts relative to a pre-set sample size* | |  |  |
|  | Minimum sample size *r* of the initial train dataset (e.g., twice the number of features of the DFM in *step 4*) | 600 [300]* |  |
| 1. *Identify newly identified eligible abstracts* | |  |  |
|  | Iterate steps 6, 7, 8 until no newly identified eligible abstracts could be identified, completed *Workflow Phase 1* |  |  |
| *Phase 2 – Figure 2 in the main article* | |  |  |
| 1. *Assemble training dataset with SVD-based features and screening results* | |  |  |
| 1. *Train random-forest model for eligibility classification* | |  |  |
|  | Train the random-forest models using the method of *k*-fold cross-validation (e.g., 10-fold) | R package ‘caret’ | ^23,46,49,50^ |
|  | Maximize sensitivity of model prediction during cross-validation |  | ^26,51^ ^50^ |
|  | Use the SMOTE algorithm to deal with imbalanced distribution in the *k* folds during cross-validation |  | ^27^ ^52^ |
| 1. *Predict eligible abstracts using the fitted random-forest model* | |  |  |
|  | Eligibility probability used in the prediction | ≥0.5 |  |
| 1. *Screen predicted eligible abstracts by pairs of reviewers, independently* | | 2 reviewers | ^48^ |
| 1. *Assess whether we still can identify new eligible abstracts* | |  |  |
|  | Use a cumulative list of eligible abstracts identified from steps of Phases 1 and 2, up to this point |  |  |
| 1. *Identify abstracts similar to each of the newly identified abstracts* | |  |  |
|  | Select the *k* nearest-neighbors (k-NN) of an eligible abstract in each of the three feature-based representation^♣^ | 15[25]* | ^47^ |
| 1. *Screen the similar abstract by pairs of reviewers, independently* | | 2 reviewers | ^48^ |

Notes: UMLS: Unified Medical Language System. DFM: document-feature matrix. Steps that are not in the above table are described in the main text, *Values used in sensitivity analysis (values are listed in squared brackets). ^♦^Citations with titles only were used in a sensitivity analysis. ^♣^The value of *k*=25 was the 75^th^ percentile of the average number of included studies in a systematic review, with a mean of 15 and the interquartile range of 8 and 25.^47^ *^•^*In our preliminary results, we observed that the upper 95% confidence interval of the estimated number of missed could be 2.6 times smaller than the actual number of missed studies (see table 3), hence we suggest to screen a number of the prioritized abstract that is equal to 3 times the upper bound of the estimated number of missed studies.

## Table B2. Performance of random-forest models with different document feature matrices

| Random-forest model with predictors based upon … | round | n.seeds | n.candidates | n.eligibles | percent | precision | recall | f1 | accuracy |
| --- | --- | --- | --- | --- | --- | --- | --- | --- | --- |
| SVD-based features | 3 | 148 | 1297 | 346 | 27 | 74% | 59% | 66% | 97% |
| Topic-modeling features | 3 | 148 | 1297 | 346 | 27 | 33% | 63% | 43% | 91% |
| Word-embedding features | 3 | 148 | 1297 | 346 | 27 | 47% | 58% | 52% | 95% |

## Step-specific results of the workflow performance – Systematic review of type 1 diabetes

### Table B3. Base case analysis

Workflow parameters: *i)* Phase 2 was conducted with SVD-based features – threshold of 70% for selecting common features, *ii)* k-nearest neighbor search with 3 distance matrices, *iii)* Initial sample size: 600, *iv)* k-NN (phase 1): 8, *v)* k-NN (phase 2): 15

| phase | round | n.seeds | n.candidates | n.eligibles | percent | precision | recall | f1-score | accuracy | tp | fp | fn | tn |
| --- | --- | --- | --- | --- | --- | --- | --- | --- | --- | --- | --- | --- | --- |
| Phase 1 | 1 | 5 | 113 | 41 | 36 |  |  |  |  |  |  |  |  |
| Phase 1 | 2 | 36 | 491 | 148 | 30 |  |  |  |  |  |  |  |  |
| Phase 1 | 3 | 107 | 1297 | 346 | 27 |  |  |  |  |  |  |  |  |
| Phase 2 | 5 |  | 1297 | 346 | 27 | 75.04% | 59.49% | 66.37% | 96.87% | 442 | 147 | 301 | 13424 |
| Phase 2 | 7 |  | 3455 | 583 | 17 | 77.68% | 80.08% | 78.86% | 97.77% | 595 | 171 | 148 | 13400 |
| Phase 2 | 9 |  | 4633 | 636 | 14 | 71.54% | 86.27% | 78.22% | 97.51% | 641 | 255 | 102 | 13316 |
| Phase 2 | 11 |  | 5052 | 650 | 13 | 70.61% | 87.62% | 78.20% | 97.46% | 651 | 271 | 92 | 13300 |
| Phase 2 | 13 |  | 5192 | 652 | 13 | 68.45% | 87.89% | 76.96% | 97.27% | 653 | 301 | 90 | 13270 |
| Phase 2 | 15 |  | 5213 | 654 | 13 | 67.60% | 88.16% | 76.52% | 97.19% | 655 | 314 | 88 | 13257 |
| Phase 2 | 17 |  | 5254 | 655 | 12 | 71.20% | 88.16% | 78.77% | 97.53% | 655 | 265 | 88 | 13306 |
| Phase 3 |  |  |  |  |  |  |  |  |  |  |  |  |  |
| rf.svd |  |  | 5254 | 655 | 12 | 71.20% | 88.16% | 78.77% | 97.53% | 655 | 265 | 88 | 13306 |
| rf.text2vec |  |  | 5254 | 655 | 12 | 65.47% | 88.56% | 75.29% | 96.98% | 658 | 347 | 85 | 13224 |
|  |  | 95% Low | 95% High | Estimate |  |  |  |  |  |  |  |  |  |
| N.missed |  | 40 | 78 | 56 |  |  |  |  |  |  |  |  |  |

Notes: rf: random forest. svd: singular-value decomposition. Text2vec: word or concept embeddings. N.seeds: number of initially known eligibles. N.candidates: candidates selected for screening. TP : true positive. FP : false positive. FN: false negative. TN: true negative.

### Table B4. Sensitivity analysis – Increasing k-nearest neighbor search in phase 2

Workflow parameters: *i)* Phase 2 was conducted with SVD-based features– threshold of 70% for selecting common features, *ii)* k-nearest neighbor search with 3 distance matrices, *iii)* Initial sample size: 600, *iv)* k-NN (phase 1): 8, *v)* **k-NN (phase 2): 25 (base value: 15)**

| round | n.seeds | n.candidates | n.eligibles | percent | precision | recall | f1 | accuracy | tp | fp | fn | tn |
| --- | --- | --- | --- | --- | --- | --- | --- | --- | --- | --- | --- | --- |
| Phase 1 | 5 | 113 | 41 | 36 |  |  |  |  |  |  |  |  |
| Phase 1 | 36 | 491 | 148 | 30 |  |  |  |  |  |  |  |  |
| Phase 1 | 107 | 1297 | 346 | 27 |  |  |  |  |  |  |  |  |
| Phase 2 |  | 1297 | 346 | 27 | 75.04% | 59.49% | 66.37% | 96.87% | 442 | 147 | 301 | 13424 |
| Phase 2 |  | 4397 | 609 | 14 | 75.33% | 83.85% | 79.36% | 97.74% | 623 | 204 | 120 | 13367 |
| Phase 2 |  | 6170 | 679 | 11 | 66.18% | 91.66% | 76.86% | 97.14% | 681 | 348 | 62 | 13223 |
| Phase 2 |  | 7014 | 695 | 10 | 63.16% | 93.67% | 75.45% | 96.84% | 696 | 406 | 47 | 13165 |
| Phase 2 |  | 7263 | 699 | 10 | 61.91% | 94.08% | 74.68% | 96.69% | 699 | 430 | 44 | 13141 |
| Phase 2 |  | 7296 | 700 | 10 | 64.94% | 94.21% | 76.88% | 97.06% | 700 | 378 | 43 | 13193 |
| Phase 2 |  | 7301 | 700 | 10 | 63.93% | 94.21% | 76.17% | 96.94% | 700 | 395 | 43 | 13176 |
| Phase 3 |  |  |  |  |  |  |  |  |  |  |  |  |
| rf.svd |  | 7301 | 700 | 10 | 63.75% | 94.21% | 76.05% | 96.92% | 700 | 398 | 43 | 13173 |
| rf.text2vec |  | 7301 | 700 | 10 | 61.35% | 94.21% | 74.31% | 96.62% | 700 | 441 | 43 | 13130 |
|  | 95% Low | 95% High | Estimate |  |  |  |  |  |  |  |  |  |
| N.missed | 65 | 113 | 86 |  |  |  |  |  |  |  |  |  |

Notes: rf: random forest. svd: singular-value decomposition. Text2vec: word or concept embeddings. N.seeds: number of initially known eligibles. N.candidates: candidates selected for screening. TP : true positive. FP : false positive. FN: false negative. TN: true negative.

### Table B5. Sensitivity analysis – Decreasing initial sample size

Workflow parameters: *i)* Phase 2 was conducted with SVD-based features – threshold of 70% for selecting common features, *ii)* k-nearest neighbor search with 3 distance matrices, ***iii)* Initial sample size: 300 (base value: 600),** *iv)* k-NN (phase 1): 8, *v)* k-NN (phase 2): 15

| phase | round | n.seeds | n.candidates | n.eligibles | percent | precision | recall | f1 | accuracy | tp | fp | fn | tn |
| --- | --- | --- | --- | --- | --- | --- | --- | --- | --- | --- | --- | --- | --- |
| Phase 1 | 1 | 5 | 113 | 41 | 36 |  |  |  |  |  |  |  |  |
| Phase 1 | 2 | 36 | 491 | 148 | 30 |  |  |  |  |  |  |  |  |
| Phase 2 | 4 |  | 491 | 148 | 30 | 54.66% | 40.24% | 46.36% | 95.17% | 299 | 248 | 444 | 13323 |
| Phase 2 | 6 |  | 2825 | 542 | 19 | 81.17% | 76.58% | 78.81% | 97.86% | 569 | 132 | 174 | 13439 |
| Phase 2 | 8 |  | 4593 | 642 | 14 | 74.36% | 86.68% | 80.05% | 97.76% | 644 | 222 | 99 | 13349 |
| Phase 2 | 10 |  | 5218 | 658 | 13 | 67.91% | 88.56% | 76.87% | 97.23% | 658 | 311 | 85 | 13260 |
| Phase 2 | 12 |  | 5403 | 660 | 12 | 68.97% | 88.83% | 77.65% | 97.35% | 660 | 297 | 83 | 13274 |
| Phase 2 | 14 |  | 5416 | 660 | 12 | 69.99% | 88.83% | 78.29% | 97.44% | 660 | 283 | 83 | 13288 |
| Phase 3 |  |  |  |  |  |  |  |  |  |  |  |  |  |
| rf.svd |  |  | 5416 | 660 | 12 | 69.92% | 88.83% | 78.25% | 97.44% | 660 | 284 | 83 | 13287 |
| rf.text2vec |  |  | 5416 | 660 | 12 | 66.30% | 88.96% | 75.98% | 97.08% | 661 | 336 | 82 | 13235 |
|  |  | 95% High | 95% Low | Estimate |  |  |  |  |  |  |  |  |  |
| N.missed |  | 38 | 74 | 53 |  |  |  |  |  |  |  |  |  |

Notes: rf: random forest. svd: singular-value decomposition. Text2vec: word or concept embeddings. N.seeds: number of initially known eligibles. N.candidates: candidates selected for screening. TP : true positive. FP : false positive. FN: false negative. TN: true negative.

### Table B6. Sensitivity analysis – Increasing k-nearest neighbor search in phase 1

Workflow parameters: *i)* Phase 2 was conducted with SVD-based features– threshold of 70% for selecting common features, *ii)* k-nearest neighbor search with 3 distance matrices, *iii)* Initial sample size: 600, ***iv)* k-NN (phase 1): 15 (base value: 8)**, *v)* k-NN (phase 2): 15

| phase | round | n.seeds | n.candidates | n.eligibles | percent | precision | recall | f1 | accuracy | tp | fp | fn | tn |
| --- | --- | --- | --- | --- | --- | --- | --- | --- | --- | --- | --- | --- | --- |
| Phase 1 | 1 | 5 | 206 | 59 | 29 |  |  |  |  |  |  |  |  |
| Phase 1 | 2 | 54 | 1075 | 282 | 26 |  |  |  |  |  |  |  |  |
| Phase 2 | 4 |  | 1075 | 282 | 26 | 63.20% | 53.16% | 57.75% | 95.96% | 395 | 230 | 348 | 13341 |
| Phase 2 | 6 |  | 3500 | 574 | 16 | 80.30% | 79.00% | 79.65% | 97.90% | 587 | 144 | 156 | 13427 |
| Phase 2 | 8 |  | 4802 | 647 | 13 | 72.93% | 87.75% | 79.66% | 97.67% | 652 | 242 | 91 | 13329 |
| Phase 2 | 10 |  | 5383 | 661 | 12 | 69.07% | 88.96% | 77.76% | 97.36% | 661 | 296 | 82 | 13275 |
| Phase 2 | 12 |  | 5507 | 662 | 12 | 71.02% | 89.37% | 79.14% | 97.55% | 664 | 271 | 79 | 13300 |
| Phase 2 | 14 |  | 5529 | 664 | 12 | 68.17% | 89.37% | 77.34% | 97.28% | 664 | 310 | 79 | 13261 |
| Phase 3 |  |  |  |  |  |  |  |  |  |  |  |  |  |
| rf.svd |  |  | 5529 | 664 | 12 | 68.31% | 89.37% | 77.43% | 97.30% | 664 | 308 | 79 | 13263 |
| rf.text2vec |  |  | 5529 | 664 | 12 | 64.56% | 89.50% | 75.01% | 96.91% | 665 | 365 | 78 | 13206 |
|  |  | 95% Low | 95% High | Estimate |  |  |  |  |  |  |  |  |  |
| n.missed |  | 46 | 86 | 63 |  |  |  |  |  |  |  |  |  |

Notes: rf: random forest. svd: singular-value decomposition. Text2vec: word or concept embeddings. N.seeds: number of initially known eligibles. N.candidates: candidates selected for screening. TP : true positive. FP : false positive. FN: false negative. TN: true negative.

### Table B7. Sensitivity analysis – Decreasing threshold for selecting common features: 80%

Workflow parameters: ***i)* Phase 2 was conducted with SVD-based features – threshold of 80% for selecting common features (base value: 70%)**, *ii)* k-nearest neighbor search with 3 distance matrices, *iii)* Initial sample size: 600, *iv)* k-NN (phase 1): 8, *v)* k-NN (phase 2): 15

| phase | round | n.seeds | n.candidates | n.eligibles | percent | precision | recall | f1 | accuracy | tp | fp | fn | tn |
| --- | --- | --- | --- | --- | --- | --- | --- | --- | --- | --- | --- | --- | --- |
| Phase 1 | 1 | 5 | 113 | 41 | 36 |  |  |  |  |  |  |  |  |
| Phase 1 | 2 | 36 | 491 | 148 | 30 |  |  |  |  |  |  |  |  |
| Phase 1 | 3 | 107 | 1297 | 346 | 27 |  |  |  |  |  |  |  |  |
| Phase 2 | 5 |  | 1297 | 346 | 27 | 76.53% | 58.82% | 66.51% | 96.93% | 437 | 134 | 306 | 13437 |
| Phase 2 | 7 |  | 3445 | 575 | 17 | 77.14% | 79.00% | 78.06% | 97.69% | 587 | 174 | 156 | 13397 |
| Phase 2 | 9 |  | 4582 | 634 | 14 | 73.59% | 85.87% | 79.25% | 97.67% | 638 | 229 | 105 | 13342 |
| Phase 2 | 11 |  | 5051 | 648 | 13 | 69.15% | 87.48% | 77.24% | 97.32% | 650 | 290 | 93 | 13281 |
| Phase 2 | 13 |  | 5200 | 651 | 13 | 70.26% | 87.75% | 78.04% | 97.44% | 652 | 276 | 91 | 13295 |
| Phase 2 | 15 |  | 5221 | 653 | 13 | 69.10% | 87.89% | 77.37% | 97.33% | 653 | 292 | 90 | 13279 |
| Phase 2 | 17 |  | 5245 | 653 | 12 | 72.15% | 87.89% | 79.25% | 97.61% | 653 | 252 | 90 | 13319 |
| Phase 3 |  |  |  |  |  |  |  |  |  |  |  |  |  |
| rf.svd |  |  | 5245 | 653 | 12 | 72.08% | 87.89% | 79.20% | 97.60% | 653 | 253 | 90 | 13318 |
| rf.text2vec |  |  | 5245 | 653 | 12 | 69.28% | 88.02% | 77.53% | 97.35% | 654 | 290 | 89 | 13281 |
|  |  | 95% Low | 95% High | Estimate |  |  |  |  |  |  |  |  |  |
| n.missed |  | 35 | 70 | 50 |  |  |  |  |  |  |  |  |  |

Notes: rf: random forest. svd: singular-value decomposition. Text2vec: word or concept embeddings. N.seeds: number of initially known eligibles. N.candidates: candidates selected for screening. TP : true positive. FP : false positive. FN: false negative. TN: true negative.

### Table B8. Sensitivity analysis – Decreasing threshold for selecting common features: 90%

Workflow parameters: ***i)* Phase 2 was conducted with SVD-based features – threshold of 90% for selecting common features (base value: 70%)**, *ii)* k-nearest neighbor search with 3 distance matrices, *iii)* Initial sample size: 600, *iv)* k-NN (phase 1): 8, *v)* k-NN (phase 2): 15

| phase | round | n.seeds | n.candidates | n.eligibles | percent | precision | recall | f1 | accuracy | tp | fp | fn | tn |
| --- | --- | --- | --- | --- | --- | --- | --- | --- | --- | --- | --- | --- | --- |
| Phase 1 | 1 | 5 | 113 | 41 | 36 |  |  |  |  |  |  |  |  |
| Phase 1 | 2 | 36 | 491 | 148 | 30 |  |  |  |  |  |  |  |  |
| Phase 1 | 3 | 107 | 1297 | 347 | 27 |  |  |  |  |  |  |  |  |
| Phase 2 | 5 |  | 1297 | 347 | 27 | 74.14% | 60.57% | 66.67% | 96.86% | 450 | 157 | 293 | 13414 |
| Phase 2 | 7 |  | 3514 | 580 | 17 | 79.44% | 79.54% | 79.49% | 97.87% | 591 | 153 | 152 | 13418 |
| Phase 2 | 9 |  | 4596 | 636 | 14 | 73.23% | 86.14% | 79.16% | 97.65% | 640 | 234 | 103 | 13337 |
| Phase 2 | 11 |  | 5068 | 649 | 13 | 70.24% | 87.35% | 77.86% | 97.42% | 649 | 275 | 94 | 13296 |
| Phase 2 | 13 |  | 5195 | 650 | 13 | 68.99% | 87.75% | 77.25% | 97.32% | 652 | 293 | 91 | 13278 |
| Phase 2 | 15 |  | 5224 | 652 | 12 | 67.63% | 87.75% | 76.39% | 97.18% | 652 | 312 | 91 | 13259 |
| Phase 3 |  |  |  |  |  |  |  |  |  |  |  |  |  |
| rf.svd |  |  | 5224 | 652 | 12 | 67.67% | 87.89% | 76.46% | 97.19% | 653 | 312 | 90 | 13259 |
| rf.text2vec |  |  | 5224 | 652 | 12 | 66.53% | 88.02% | 75.78% | 97.08% | 654 | 329 | 89 | 13242 |
|  |  | 95% Low | 95% High | Estimate |  |  |  |  |  |  |  |  |  |
| n.missed |  | 42 | 80 | 58 |  |  |  |  |  |  |  |  |  |

Notes: rf: random forest. svd: singular-value decomposition. Text2vec: word or concept embeddings. N.seeds: number of initially known eligibles. N.candidates: candidates selected for screening. TP : true positive. FP : false positive. FN: false negative. TN: true negative.

### Table B9. Sensitivity analysis – No distance matrix for topic-modeling-based features

Workflow parameters: *i)* Phase 2 was conducted with SVD-based features– threshold of 70% for selecting common features, *ii)* **k-nearest neighbor search with 2 distance matrices (base value: 3 distance matrices)**, *iii)* Initial sample size: 600, *iv)* k-NN (phase 1): 8, *v)* k-NN (phase 2): 15

| phase | round | n.seeds | n.candidates | n.eligibles | percent | precision | recall | f1 | accuracy | tp | fp | fn | tn |
| --- | --- | --- | --- | --- | --- | --- | --- | --- | --- | --- | --- | --- | --- |
| Phase 1 | 1 | 5 | 80 | 35 | 44 |  |  |  |  |  |  |  |  |
| Phase 1 | 2 | 30 | 307 | 104 | 34 |  |  |  |  |  |  |  |  |
| Phase 1 | 3 | 69 | 686 | 243 | 35 |  |  |  |  |  |  |  |  |
| Phase 2 | 5 |  | 686 | 243 | 35 | 65.70% | 48.72% | 55.95% | 96.02% | 362 | 189 | 381 | 13382 |
| Phase 2 | 7 |  | 2085 | 502 | 24 | 84.46% | 68.78% | 75.82% | 97.72% | 511 | 94 | 232 | 13477 |
| Phase 2 | 9 |  | 2928 | 563 | 19 | 82.59% | 77.25% | 79.83% | 97.97% | 574 | 121 | 169 | 13450 |
| Phase 2 | 11 |  | 3334 | 598 | 18 | 80.75% | 80.75% | 80.75% | 98.00% | 600 | 143 | 143 | 13428 |
| Phase 2 | 13 |  | 3499 | 606 | 17 | 79.63% | 81.56% | 80.59% | 97.96% | 606 | 155 | 137 | 13416 |
| Phase 2 | 15 |  | 3581 | 611 | 17 | 80.34% | 82.50% | 81.41% | 98.04% | 613 | 150 | 130 | 13421 |
| Phase 2 | 17 |  | 3679 | 617 | 17 | 78.33% | 83.18% | 80.68% | 97.93% | 618 | 171 | 125 | 13400 |
| Phase 2 | 19 |  | 3741 | 619 | 17 | 78.45% | 83.31% | 80.81% | 97.95% | 619 | 170 | 124 | 13401 |
| Phase 2 | 21 |  | 3752 | 620 | 17 | 76.54% | 83.85% | 80.03% | 97.83% | 623 | 191 | 120 | 13380 |
| Phase 3 |  |  |  |  |  |  |  |  |  |  |  |  |  |
| rf.svd |  |  | 3778 | 625 | 17 | 76.63% | 83.85% | 80.08% | 97.83% | 623 | 190 | 120 | 13381 |
| rf.text2vec |  |  | 3778 | 625 | 17 | 71.90% | 84.39% | 77.65% | 97.48% | 627 | 245 | 116 | 13326 |
|  |  | 95% Low | 95% High | Estimate |  |  |  |  |  |  |  |  |  |
| N.missed |  | 39 | 76 | 55 |  |  |  |  |  |  |  |  |  |

Notes: rf: random forest. svd: singular-value decomposition. Text2vec: word or concept embeddings. N.seeds: number of initially known eligibles. N.candidates: candidates selected for screening. TP : true positive. FP : false positive. FN: false negative. TN: true negative.

## Step-specific results of the workflow performance – Scoping review of knowledge-synthesis methods

### Table B10. Base case analysis

Workflow parameters: *i)* Phase 2 was conducted with SVD-based features – threshold of 70% for selecting common features, *ii)* k-nearest neighbor search with 3 distance matrices, *iii)* Initial sample size: 600, *iv)* k-NN (phase 1): 8, *v)* k-NN (phase 2): 15

| phase | round | n.seeds | n.candidates | n.eligibles | percent | precision | recall | f1 | accuracy | tp | fp | fn | tn |
| --- | --- | --- | --- | --- | --- | --- | --- | --- | --- | --- | --- | --- | --- |
| Phase 1 | 1 | 4 | 63 | 47 | 75 |  |  |  |  |  |  |  |  |
| Phase 1 | 2 | 43 | 444 | 170 | 38 |  |  |  |  |  |  |  |  |
| Phase 1 | 3 | 123 | 1454 | 370 | 25 |  |  |  |  |  |  |  |  |
| Phase 2 | 5 |  | 1454 | 370 | 25 | 81.31% | 50.47% | 62.28% | 96.60% | 483 | 111 | 474 | 16132 |
| Phase 2 | 7 |  | 4450 | 706 | 16 | 78.52% | 75.24% | 76.84% | 97.48% | 720 | 197 | 237 | 16046 |
| Phase 2 | 9 |  | 6318 | 801 | 13 | 76.45% | 84.12% | 80.10% | 97.67% | 805 | 248 | 152 | 15995 |
| Phase 2 | 11 |  | 7101 | 830 | 12 | 74.82% | 86.94% | 80.43% | 97.65% | 832 | 280 | 125 | 15963 |
| Phase 2 | 13 |  | 7452 | 836 | 11 | 73.28% | 87.98% | 79.96% | 97.55% | 842 | 307 | 115 | 15936 |
| Phase 2 | 15 |  | 7632 | 845 | 11 | 71.42% | 88.51% | 79.05% | 97.39% | 847 | 339 | 110 | 15904 |
| Phase 2 | 17 |  | 7709 | 849 | 11 | 71.69% | 88.92% | 79.38% | 97.43% | 851 | 336 | 106 | 15907 |
| Phase 2 | 19 |  | 7737 | 852 | 11 | 71.54% | 89.03% | 79.33% | 97.42% | 852 | 339 | 105 | 15904 |
| Phase 3 |  |  |  |  |  |  |  |  |  |  |  |  |  |
| rf.svd |  |  | 7753 | 852 | 11 | 71.66% | 89.03% | 79.40% | 97.43% | 852 | 337 | 105 | 15906 |
| rf.text2vec |  |  | 7753 | 852 | 11 | 63.17% | 89.45% | 74.05% | 96.51% | 856 | 499 | 101 | 15744 |
|  |  | 95% Low | 95% High | Estimate |  |  |  |  |  |  |  |  |  |
| n.missed |  | 82 | 136 | 106 |  |  |  |  |  |  |  |  |  |

Notes: rf: random forest. svd: singular-value decomposition. Text2vec: word or concept embeddings. N.seeds: number of initially known eligibles. N.candidates: candidates selected for screening. TP : true positive. FP : false positive. FN: false negative. TN: true negative.

### Table B11. Sensitivity analysis – Increasing k-nearest neighbor search in phase 2

Workflow parameters: *i)* Phase 2 was conducted with SVD-based features– threshold of 70% for selecting common features, *ii)* k-nearest neighbor search with 3 distance matrices, *iii)* Initial sample size: 600, *iv)* k-NN (phase 1): 8, *v)* **k-NN (phase 2): 25 (base value: 15)**

| phase | round | n.seeds | n.candidates | n.eligibles | percent | precision | recall | f1 | accuracy | tp | fp | fn | tn |
| --- | --- | --- | --- | --- | --- | --- | --- | --- | --- | --- | --- | --- | --- |
| Phase 1 | 1 | 4 | 63 | 47 | 75 |  |  |  |  |  |  |  |  |
| Phase 1 | 2 | 43 | 444 | 170 | 38 |  |  |  |  |  |  |  |  |
| Phase 1 | 3 | 123 | 1454 | 370 | 25 |  |  |  |  |  |  |  |  |
| Phase 2 | 5 |  | 1454 | 370 | 25 | 81.31% | 50.47% | 62.28% | 96.60% | 483 | 111 | 474 | 16132 |
| Phase 2 | 7 |  | 5651 | 758 | 13 | 78.46% | 80.67% | 79.55% | 97.69% | 772 | 212 | 185 | 16031 |
| Phase 2 | 9 |  | 8523 | 862 | 10 | 69.80% | 90.80% | 78.93% | 97.30% | 869 | 376 | 88 | 15867 |
| Phase 2 | 11 |  | 9789 | 894 | 9 | 67.98% | 93.42% | 78.70% | 97.19% | 894 | 421 | 63 | 15822 |
| Phase 2 | 13 |  | 10167 | 897 | 9 | 66.05% | 93.94% | 77.57% | 96.98% | 899 | 462 | 58 | 15781 |
| Phase 2 | 15 |  | 10289 | 899 | 9 | 66.11% | 94.36% | 77.74% | 96.99% | 903 | 463 | 54 | 15780 |
| Phase 2 | 17 |  | 10346 | 904 | 9 | 66.33% | 94.67% | 78.00% | 97.03% | 906 | 460 | 51 | 15783 |
| Phase 2 | 19 |  | 10430 | 907 | 9 | 65.16% | 94.78% | 77.22% | 96.89% | 907 | 485 | 50 | 15758 |
| Phase 3 |  |  |  |  |  |  |  |  |  |  |  |  |  |
| rf.svd |  |  | 10456 | 907 | 9 | 65.25% | 94.78% | 77.29% | 96.90% | 907 | 483 | 50 | 15760 |
| rf.text2vec |  |  | 10456 | 907 | 9 | 62.68% | 94.78% | 75.46% | 96.57% | 907 | 540 | 50 | 15703 |
|  |  | 95% Low | 95% High | Estimate |  |  |  |  |  |  |  |  |  |
| n.missed |  | 102 | 162 | 129 |  |  |  |  |  |  |  |  |  |

Notes: rf: random forest. svd: singular-value decomposition. Text2vec: word or concept embeddings. N.seeds: number of initially known eligibles. N.candidates: candidates selected for screening. TP : true positive. FP : false positive. FN: false negative. TN: true negative.

### Table B12. Sensitivity analysis – Decreasing initial sample size

Workflow parameters: *i)* Phase 2 was conducted with SVD-based features – threshold of 70% for selecting common features, *ii)* k-nearest neighbor search with 3 distance matrices, ***iii)* Initial sample size: 300 (base value: 600),** *iv)* k-NN (phase 1): 8, *v)* k-NN (phase 2): 15

| phase | round | n.seeds | n.candidates | n.eligibles | percent | precision | recall | f1 | accuracy | tp | fp | fn | tn |
| --- | --- | --- | --- | --- | --- | --- | --- | --- | --- | --- | --- | --- | --- |
| Phase 1 | 1 | 4 | 63 | 47 | 75 |  |  |  |  |  |  |  |  |
| Phase 1 | 2 | 43 | 444 | 170 | 38 |  |  |  |  |  |  |  |  |
| Phase 2 | 4 |  | 444 | 170 | 38 | 68.78% | 40.75% | 51.18% | 95.67% | 390 | 177 | 567 | 16066 |
| Phase 2 | 6 |  | 4057 | 676 | 17 | 77.61% | 73.15% | 75.31% | 97.33% | 700 | 202 | 257 | 16041 |
| Phase 2 | 8 |  | 6289 | 793 | 13 | 74.26% | 83.49% | 78.60% | 97.47% | 799 | 277 | 158 | 15966 |
| Phase 2 | 10 |  | 7112 | 831 | 12 | 72.90% | 87.15% | 79.39% | 97.48% | 834 | 310 | 123 | 15933 |
| Phase 2 | 12 |  | 7524 | 842 | 11 | 71.71% | 88.19% | 79.10% | 97.41% | 844 | 333 | 113 | 15910 |
| Phase 2 | 14 |  | 7689 | 846 | 11 | 70.81% | 88.71% | 78.76% | 97.34% | 849 | 350 | 108 | 15893 |
| Phase 2 | 16 |  | 7803 | 851 | 11 | 71.46% | 89.24% | 79.37% | 97.42% | 854 | 341 | 103 | 15902 |
| Phase 2 | 18 |  | 7884 | 855 | 11 | 72.05% | 89.45% | 79.81% | 97.48% | 856 | 332 | 101 | 15911 |
| Phase 2 | 20 |  | 7935 | 858 | 11 | 71.74% | 89.66% | 79.70% | 97.46% | 858 | 338 | 99 | 15905 |
| Phase 3 |  |  |  |  |  |  |  |  |  |  |  |  |  |
| rf.svd |  |  | 7982 | 859 | 11 | 71.56% | 89.66% | 79.59% | 97.44% | 858 | 341 | 99 | 15902 |
| rf.text2vec | |  | 7982 | 859 | 11 | 68.88% | 89.97% | 78.02% | 97.18% | 861 | 389 | 96 | 15854 |
|  |  | 95% Low | 95% High | Estimate |  |  |  |  |  |  |  |  |  |
| n.missed |  | 69 | 118 | 91 |  |  |  |  |  |  |  |  |  |

Notes: rf: random forest. svd: singular-value decomposition. Text2vec: word or concept embeddings. N.seeds: number of initially known eligibles. N.candidates: candidates selected for screening. TP : true positive. FP : false positive. FN: false negative. TN: true negative.

### Table B13. Sensitivity analysis – Increasing k-nearest neighbor search in phase 1

Workflow parameters: *i)* Phase 2 was conducted with SVD-based features– threshold of 70% for selecting common features, *ii)* k-nearest neighbor search with 3 distance matrices, *iii)* Initial sample size: 600, ***iv)* k-NN (phase 1): 15 (base value: 8)**, *v)* k-NN (phase 2): 15

| phase | round | n.seeds | n.candidates | n.eligibles | percent | precision | recall | f1 | accuracy | tp | fp | fn | tn |
| --- | --- | --- | --- | --- | --- | --- | --- | --- | --- | --- | --- | --- | --- |
| Phase 1 | 1 | 4 | 115 | 75 | 65 |  |  |  |  |  |  |  |  |
| Phase 1 | 2 | 71 | 1034 | 294 | 28 |  |  |  |  |  |  |  |  |
| Phase 2 | 4 |  | 1034 | 294 | 28 | 74.02% | 47.34% | 57.74% | 96.15% | 453 | 159 | 504 | 16084 |
| Phase 2 | 6 |  | 4797 | 722 | 15 | 75.80% | 76.91% | 76.35% | 97.35% | 736 | 235 | 221 | 16008 |
| Phase 2 | 8 |  | 6705 | 815 | 12 | 74.23% | 85.48% | 79.46% | 97.54% | 818 | 284 | 139 | 15959 |
| Phase 2 | 10 |  | 7399 | 840 | 11 | 72.75% | 87.88% | 79.60% | 97.49% | 841 | 315 | 116 | 15928 |
| Phase 2 | 12 |  | 7667 | 842 | 11 | 74.51% | 87.98% | 80.69% | 97.66% | 842 | 288 | 115 | 15955 |
| Phase 2 | 14 |  | 7671 | 842 | 11 | 73.97% | 88.19% | 80.46% | 97.62% | 844 | 297 | 113 | 15946 |
| Phase 2 | 16 |  | 7709 | 844 | 11 | 72.27% | 88.51% | 79.57% | 97.47% | 847 | 325 | 110 | 15918 |
| Phase 2 | 18 |  | 7744 | 849 | 11 | 72.77% | 88.82% | 80.00% | 97.53% | 850 | 318 | 107 | 15925 |
| Phase 2 | 20 |  | 7805 | 851 | 11 | 72.47% | 89.13% | 79.94% | 97.51% | 853 | 324 | 104 | 15919 |
| Phase 3 |  |  |  |  |  |  |  |  |  |  |  |  |  |
| rf.svd |  |  | 7849 | 853 | 11 | 72.53% | 89.13% | 79.98% | 97.52% | 853 | 323 | 104 | 15920 |
| rf.text2vec |  |  | 7849 | 853 | 11 | 69.48% | 89.45% | 78.21% | 97.23% | 856 | 376 | 101 | 15867 |
|  |  | 95% Low | 95% High | Estimate |  |  |  |  |  |  |  |  |  |
| n.missed |  | 61 | 107 | 81 |  |  |  |  |  |  |  |  |  |

Notes: rf: random forest. svd: singular-value decomposition. Text2vec: word or concept embeddings. N.seeds: number of initially known eligibles. N.candidates: candidates selected for screening. TP : true positive. FP : false positive. FN: false negative. TN: true negative.

### Table B14. Sensitivity analysis – Decreasing threshold for selecting common features: 80%

Workflow parameters: ***i)* Phase 2 was conducted with SVD-based features – threshold of 80% for selecting common features (base value: 70%)**, *ii)* k-nearest neighbor search with 3 distance matrices, *iii)* Initial sample size: 600, *iv)* k-NN (phase 1): 8, *v)* k-NN (phase 2): 15

| phase | round | n.seeds | n.candidates | n.eligibles | percent | precision | recall | f1 | accuracy | tp | fp | fn | tn |
| --- | --- | --- | --- | --- | --- | --- | --- | --- | --- | --- | --- | --- | --- |
| Phase 1 | 1 | 4 | 63 | 47 | 75 |  |  |  |  |  |  |  |  |
| Phase 1 | 2 | 43 | 443 | 169 | 38 |  |  |  |  |  |  |  |  |
| Phase 1 | 3 | 122 | 1447 | 365 | 25 |  |  |  |  |  |  |  |  |
| Phase 2 | 5 |  | 1447 | 365 | 25 | 82.48% | 50.68% | 62.78% | 96.66% | 485 | 103 | 472 | 16140 |
| Phase 2 | 7 |  | 4564 | 708 | 16 | 78.83% | 75.86% | 77.32% | 97.52% | 726 | 195 | 231 | 16048 |
| Phase 2 | 9 |  | 6404 | 807 | 13 | 76.61% | 84.54% | 80.38% | 97.70% | 809 | 247 | 148 | 15996 |
| Phase 2 | 11 |  | 7129 | 832 | 12 | 73.25% | 87.25% | 79.64% | 97.52% | 835 | 305 | 122 | 15938 |
| Phase 2 | 13 |  | 7479 | 840 | 11 | 69.64% | 88.19% | 77.82% | 97.20% | 844 | 368 | 113 | 15875 |
| Phase 2 | 15 |  | 7677 | 847 | 11 | 71.96% | 88.51% | 79.38% | 97.44% | 847 | 330 | 110 | 15913 |
| Phase 3 |  |  |  |  |  |  |  |  |  |  |  |  |  |
| rf.svd |  |  | 7727 | 849 | 11 | 72.02% | 88.51% | 79.42% | 97.45% | 847 | 329 | 110 | 15914 |
| rf.text2vec |  |  | 7727 | 849 | 11 | 64.28% | 89.13% | 74.69% | 96.64% | 853 | 474 | 104 | 15769 |
|  |  | 95% Low | 95% High | Estimate |  |  |  |  |  |  |  |  |  |
| n.missed |  | 77 | 130 | 101 |  |  |  |  |  |  |  |  |  |

Notes: rf: random forest. svd: singular-value decomposition. Text2vec: word or concept embeddings. N.seeds: number of initially known eligibles. N.candidates: candidates selected for screening. TP : true positive. FP : false positive. FN: false negative. TN: true negative.

### Table B15. Sensitivity analysis – Decreasing threshold for selecting common features: 90%

Workflow parameters: ***i)* Phase 2 was conducted with SVD-based features – threshold of 90% for selecting common features (base value: 70%)**, *ii)* k-nearest neighbor search with 3 distance matrices, *iii)* Initial sample size: 600, *iv)* k-NN (phase 1): 8, *v)* k-NN (phase 2): 15

| phase | round | n.seeds | n.candidates | n.eligibles | percent | precision | recall | f1 | accuracy | tp | fp | fn | tn |
| --- | --- | --- | --- | --- | --- | --- | --- | --- | --- | --- | --- | --- | --- |
| Phase 1 | 1 | 4 | 63 | 47 | 75 |  |  |  |  |  |  |  |  |
| Phase 1 | 2 | 43 | 443 | 169 | 38 |  |  |  |  |  |  |  |  |
| Phase 1 | 3 | 122 | 1447 | 367 | 25 |  |  |  |  |  |  |  |  |
| Phase 2 | 5 |  | 1447 | 367 | 25 | 84.62% | 49.43% | 62.40% | 96.69% | 473 | 86 | 484 | 16157 |
| Phase 2 | 7 |  | 4292 | 695 | 16 | 78.06% | 74.71% | 76.35% | 97.42% | 715 | 201 | 242 | 16042 |
| Phase 2 | 9 |  | 6291 | 803 | 13 | 73.63% | 84.33% | 78.62% | 97.45% | 807 | 289 | 150 | 15954 |
| Phase 2 | 11 |  | 7109 | 830 | 12 | 71.36% | 86.94% | 78.38% | 97.33% | 832 | 334 | 125 | 15909 |
| Phase 2 | 13 |  | 7421 | 835 | 11 | 72.14% | 87.67% | 79.15% | 97.43% | 839 | 324 | 118 | 15919 |
| Phase 2 | 15 |  | 7554 | 841 | 11 | 72.46% | 87.98% | 79.47% | 97.47% | 842 | 320 | 115 | 15923 |
| Phase 3 |  |  |  |  |  |  |  |  |  |  |  |  |  |
| rf.svd |  |  | 7615 | 843 | 11 | 72.52% | 87.98% | 79.51% | 97.48% | 842 | 319 | 115 | 15924 |
| rf.text2vec |  |  | 7615 | 843 | 11 | 65.66% | 88.71% | 75.47% | 96.79% | 849 | 444 | 108 | 15799 |
|  |  | 95% Low | 95% High | Estimate |  |  |  |  |  |  |  |  |  |
| n.missed |  | 74 | 125 | 97 |  |  |  |  |  |  |  |  |  |

Notes: rf: random forest. svd: singular-value decomposition. Text2vec: word or concept embeddings. N.seeds: number of initially known eligibles. N.candidates: candidates selected for screening. TP : true positive. FP : false positive. FN: false negative. TN: true negative.

### Table B16. Sensitivity analysis – No distance matrix for topic-modeling-based features

Workflow parameters: *i)* Phase 2 was conducted with SVD-based features– threshold of 70% for selecting common features, *ii)* **k-nearest neighbor search with 2 distance matrices (base value: 3 distance matrices)**, *iii)* Initial sample size: 600, *iv)* k-NN (phase 1): 8, *v)* k-NN (phase 2): 15

| phase | round | n.seeds | n.candidates | n.eligibles | percent | precision | recall | f1 | accuracy | tp | fp | fn | tn |
| --- | --- | --- | --- | --- | --- | --- | --- | --- | --- | --- | --- | --- | --- |
| Phase 1 | 1 | 4 | 53 | 37 | 70 |  |  |  |  |  |  |  |  |
| Phase 1 | 2 | 33 | 277 | 128 | 46 |  |  |  |  |  |  |  |  |
| Phase 1 | 3 | 91 | 780 | 265 | 34 |  |  |  |  |  |  |  |  |
| Phase 2 | 5 |  | 780 | 265 | 34 | 71.75% | 46.19% | 56.20% | 95.99% | 442 | 174 | 515 | 16069 |
| Phase 2 | 7 |  | 3159 | 638 | 20 | 81.12% | 68.23% | 74.12% | 97.35% | 653 | 152 | 304 | 16091 |
| Phase 2 | 9 |  | 4415 | 712 | 16 | 76.93% | 74.92% | 75.91% | 97.35% | 717 | 215 | 240 | 16028 |
| Phase 2 | 11 |  | 4829 | 753 | 16 | 77.57% | 79.52% | 78.53% | 97.58% | 761 | 220 | 196 | 16023 |
| Phase 2 | 13 |  | 5159 | 777 | 15 | 77.78% | 81.19% | 79.45% | 97.66% | 777 | 222 | 180 | 16021 |
| Phase 2 | 15 |  | 5282 | 783 | 15 | 74.81% | 81.92% | 78.20% | 97.46% | 784 | 264 | 173 | 15979 |
| Phase 2 | 17 |  | 5331 | 784 | 15 | 75.70% | 82.03% | 78.74% | 97.53% | 785 | 252 | 172 | 15991 |
| Phase 2 | 19 |  | 5335 | 785 | 15 | 78.58% | 82.03% | 80.27% | 97.76% | 785 | 214 | 172 | 16029 |
| Phase 3 |  |  |  |  |  |  |  |  |  |  |  |  |  |
| rf.svd |  |  | 5335 | 785 | 15 | 78.58% | 82.03% | 80.27% | 97.76% | 785 | 214 | 172 | 16029 |
| rf.text2vec |  |  | 5335 | 785 | 15 | 70.89% | 82.97% | 76.46% | 97.16% | 794 | 326 | 163 | 15917 |
|  |  | 95% Low | 95% High | Estimate |  |  |  |  |  |  |  |  |  |
| n.missed |  | 32 | 65 | 46 |  |  |  |  |  |  |  |  |  |

Notes: rf: random forest. svd: singular-value decomposition. Text2vec: word or concept embeddings. N.seeds: number of initially known eligibles. N.candidates: candidates selected for screening. TP : true positive. FP : false positive. FN: false negative. TN: true negative.

# Appendix C: Workflow implementation in R codes

# Workflow of 11 steps as described in Pham et al. Text mining to support abstract screening for systematic reviews: A workflow approach

# The R codes were adopted from Jelena Jovanovic, LASI'18 Workshop on Text mining for learning content analysis

# https://github.com/jeljov/Text_Mining_at_LASI18

# Codes were written in R using RStudio. Recommended latest version of RStudio and R.

# See "Humans and computing resources required for implementing the workflow" in the online Appendix of the article.

# Required packages for Figure 1 in the main paper.

# Figure.1.packages = c("dplyr", "tidyr", "caret", "rpart", "e1071", "DMwR", "quanteda", "kernlab", "randomForest", "nnet",

# "rpart.plot", "klaR", "irlba", "doSNOW","text2vec","glmnet","udpipe","topicmodels","purr")

# install.packages(Figure.1.packages) # To only install once for R installation (and update). To attach the package libraries for each R session.

# rm(list = ls()) # clean up all previous objects

# Step 0. Attach the library

library(readr) # tools to read rectangular data (like 'csv', 'tsv', and 'fwf')

library(dplyr) # tools for working with R data frames

library(tidyr) # tools to tidy R codes

library(caret) # Classification And REgression Training

library(rpart) # Recursive PARTitioning for building Classification and Regression Trees

library(e1071) # tools for support vector machines and many other topics

library(DMwR) # data mining tools

library(kernlab) # tools for kernel-based machine learning methods for classification,regression

library(quanteda) # tools for managing and analyzing textual data

library(randomForest) # tools for building random forest for classification

library(irlba) # tools for Fast Truncated Singular Value Decomposition and Principal Components Analysis

library(stringr) # tools for character manipulation

library(doSNOW) # parallel processing

library(text2vec) # text analysis and natural language processing (e.g., dist2)

# library(nnet) # neural network model

library(udpipe) # NLP language models

library(topicmodels) # topic modeling, including Latent Dirichlet Allocation model

library(purrr) # tools for working with functions and vectors

# Initialization

pos.label="INCLUDE" # coding of screening results

neg.label="EXCLUDE"

###################################################################################

# Step 1. Import citations (Figure 1)

## Import input data of abstract text and results of abstract screening by human reviewers into R

## Input data: Comma-separated values (CSV) file with column names: id, title, abstract, status. See the file sample and some explanations below.

## column "id": numerical id of abstracts. Each abstract appears twice, as it was reviewed by pairs of reviewers.

## columns "title", "abstract": title and abstract of each abstract

## column "status": results of abstract screening by human reviewers, such as "INCLUDED" or "EXCLUDED"

## For status, use uppercase in the coding of "INCLUDED" or "EXCLUDED"; otherwise, please find and replace the hard coding categories above.

## In our practice, we put the protocol as the first record in the corpus as it contains all the relevant terms for abstract screening

# raw.file="/home/ba/ranking/csvfiles/sr t2d final.csv"

raw.file="/home/ba/ranking/csvfiles/ks methods.csv"

raw.ta=read.csv(file=raw.file,header=TRUE,stringsAsFactors=FALSE)

raw.ta=raw.ta[,-c(4,5,6)] # exclude the question 1-3 results

## check and remove abstracts with missing status

jid=which(raw.ta$status=="") # retrieve row number of abstracts with missing screening results

raw.ta=raw.ta[-jid,] # remove these abstracts

## The input data is organized as screening results (after reconciliation) from two reviewers

## Each abstract is represented by two rows, for the first and second reviewers, but the screening results are the same after reconciliation

selected.ta= seq(from=1, to=nrow(raw.ta) - 1, by=2)

## keep data from one reviewer only in the working TA's database

wkta=raw.ta[selected.ta,]

rm(raw.ta) # remove unused R datasets from the R space

# coding the text categories of the screening results into R categories

wkta[,4] = factor(wkta[,4],levels=c(pos.label,neg.label)) # machine-learning models to predict the first category of included abstracts

summary(wkta[,4])

### merge titles and abstracts into text for analysis, retain variables in wkta

wkta=wkta %>% mutate(text=paste(title,abstract,sep=". ")) %>% dplyr::select(id,title,abstract,text,status)

dim(wkta)

###################################################################################

# Step 2. Pre-process text (Figure 1)

###################################################################################

## Take a look at a specific abstract and observe the "copyright" info

display_id=662067 # this abstract is with copyright info

wkta$text[wkta$id==display_id] # display th etext, see Copyright info at the end of the text

## A regular expression is a special text string for describing a search pattern.

## See for example a quick reference and an online testing tool for building your regular expressions

## https://www.regexbuddy.com/regex.html

## https://regex101.com/

## Regular expression to locate "Copy right" information in an abstract.

## Note: in R, you need to double the "\\" in the specification of the regular expression.

copyright.pattern = "\\bCopyright\\b(.)*" # match the word and any characters beyond to the end of the abstract

## remove all "copyright" info from text

jx = wkta$text %>% purrr::map(sub,pattern=copyright.pattern,replacement="",ignore.case=TRUE) # tidyR format

jy = sub(pattern=copyright.pattern,replacement="",x=wkta$text,ignore.case=TRUE) # old format

jx[wkta$id==display_id] # check if copyright info in abstract 662067 is gone

wkta$text=unlist(jx) # remove [[]] to [] # now all copyright info were removed

###############################################################################################

# Step 3. Construct features for singular value decomposition: 1-, 2, 3-gram phrases (Figure 1)

###############################################################################################

## Tokenize the text while removing numbers, punctuation marks, symbols, and so on

svd.tokens <- quanteda::tokens(x = wkta$text, what = "word", remove_numbers = TRUE,remove_punct = TRUE,

remove_symbols = TRUE, remove_hyphens= FALSE, ngrams=1:3,

concatenator="_") # including single words, and two-word and three-word word-phrases

## if needed, see "tokenization" in the Glossary, online Appendix

str(svd.tokens) # check the volume of tokens

## remove tokens with 1 or 2 characters only as they rarely bear any meaning

svd.tokens <- tokens_keep(x = svd.tokens, min_nchar = 3)

## to lower letter

svd.tokens <- tokens_tolower(svd.tokens)

## remove stopwords (if needed, see "pre-process text" in Glossary, online Appendix)

head(stopwords(), n = 20) # display the first 20 stopwords in a total of 175 words

svd.tokens <- tokens_remove(svd.tokens, stopwords())

## Perform stemming on the tokens (if needed, see "pre-process text" in Glossary, online Appendix)

svd.tokens <- tokens_wordstem(svd.tokens, language = "english")

## create Document-Feature Matrix with Term frequencies (TF) - Inverse Document Frequencies (IDF) as feature weights

## if needed, see "feature construction" and related terms in Glossary, online Appendix

svd.dfm <- dfm(x = svd.tokens, tolower = FALSE) %>% dfm_tfidf(scheme_tf = "prop")

dim(svd.dfm) # number of terms from the corpus, typically a few million terms

##################################################################################

## Step 4. Select features (Figure 1)

## Step 4a. Select features based upon selected threshold of feature weights

## Step 4b. Use singular value decomposition for further dimension reduction (see Glossary, online Appendix)

## Step 4c. Retain 300 features which are linear combinations of the selected features

##################################################################################

## Step 4a. Select features based upon selected threshold of feature weights

### Examine the distribution of the feature weights.

features.wts=colSums(svd.dfm) # each feature weight is the sum of the weights of the feature across abstracts

summary(features.wts) # inspect the distribution of weights of features in the corpus

### set threshold to keep only features with significant feature weights in the corpus (see Table 2 in the main paper)

## keep features with weight above the quantile of 0.7, 0.8, 0.9 of the feature weight distribution (Table 2)

threshold.p = 0.90

threshold.wts <- quantile(features.wts, probs = threshold.p)

threshold.wts

to_keep <- which(features.wts > threshold.wts) # indices of selected features

### keep features with weights above threshold of weight distribution in the document-feature matrix

svd.dfm1 <- dfm_keep(svd.dfm, pattern = names(to_keep), valuetype = "fixed", verbose = TRUE) # matrix with TF-IDF for SVD

### inspect the number of vocabulary

nc.retained.features=dim(svd.dfm1) # ~702K terms for case study 1

topfeatures(svd.dfm1,n=50) # inspect top features with highest feature weights

jx=tail(topfeatures(svd.dfm1, n =ncol(svd.dfm1)), n=100) # inspect the terms that are at the tail of the distribution

attributes(jx)$names

## Step 4b. Use Singular Value Decomposition (SVD, if needed, see Glossary, online Appendix) for dimension reduction

### Obtain 300 linear combinations of selected features (see supporting evidence for the value of 300 in Table 2)

start.time <- Sys.time() # this is time consuming of up to 8 hours for a threshold of 70%

# cat(" HERE IS WHERE WE START: ", format(Sys.time(), "%a %b %d %X %Y"), "\n")

my.cluster <- makeCluster(spec=6, type = "SOCK") # parallel processing

registerDoSNOW(my.cluster)

sr.svd <- irlba(t(svd.dfm1), # it is transposed as SVD requires Term-Document Matrix as an input

nv = 300, # number of singular vectors to estimate

maxit = 600) # maxit is recommended to be twice larger than nv

stopCluster(my.cluster)

svd.time=Sys.time() - start.time ## keep track of this.

## cat("Computing time for SVD: ", Sys.time() - start.time, "\n")

## system("echo \"All done\" | mailx -s \'Complete SVD \' ba.pham@theta.utoronto.ca")

### Reduced Document-Features Matrix for ML classification

sr.svd.X = sr.svd$v # Reduced DFM of 'n' abstracts x 300 features

dim(sr.svd.X)

## saveRDS(sr.svd.X, "scopingr_svd90.RData") # threshold of 80%

## saveRDS(sr.svd.X, "scopingr_svd80.RData") # threshold of 80%

## X matrix from SVD at differenct cutoff of feature weights

# saveRDS(sr.svd.X, "scopingr_svd70.RData") # threshold of 70%

# saveRDS(sr.svd.X, "sr_svd80.RData") # threshold of 80%

# saveRDS(sr.svd.X, "sr_svd90.RData") # threshold of 90%

# sr.svd.X <- readRDS("sr_svd70.RData")

# sr.svd.X <- readRDS("scopingr_svd70.RData")

###########################################################################################################################

## Steps 2-4. Build additional feature matrix to supplement the feature matrix from Singular Value Decomposition (Figure 1)

## Step 2. Natural language processing of abstracts to extract nouns and verbs

## Step 3. Build document-feature matrix of lemmatized nouns and verbs

## Step 4a. Conduct topic modeling with Latent Dirichlet Allocation

## Step 4b. Obtain the posterior topic distributions of abstracts to be used as feature matrix with 300 topics

###########################################################################################################################

## Step 2 - use the *udpipe* R package

## https://github.com/bnosac/udpipe

my.dir="/home/ba/ranking/csvfiles" # storage

language.model="/home/ba/ranking/csvfiles/english-ud-2.0-170801.udpipe"

## Load the appropriate language model (the one for English language)

tagger <- udpipe_download_model("english", model_dir = my.dir)

tagger <- udpipe_load_model(file = language.model)

## Annotate the text of the abstracts using the loaded model (tagger).

## This will produce several linguistic annotations for each word, including the appropriate POS tags and lemmas

start.time <- Sys.time()

my.cluster <- makeCluster(spec=6, type = "SOCK") # parallel processing

registerDoSNOW(my.cluster)

abstract_annotated <- udpipe_annotate(tagger, wkta$text, doc_id =wkta$id) # this step is time consuming, about 2 hours

stopCluster(my.cluster)

## To be able to use the udpipe object easily, we'll transform it into a data frame, see Jelena Jovanovic, LASI'18

abst.ann.df <- as.data.frame(abstract_annotated) # Notes: udpipe provides words and their lemma's

## Save the object to have it available for later

saveRDS(abst.ann.df, "scopingr_abst_ann_df.RData")

# abst.ann.df <- readRDS("abst_ann_df.RData")

## and remove the large udpipe object, to release memory * BA is here

remove(abstract_annotated)

abstract.annotated.time=Sys.time() - start.time ## keep track of computing time

## check the total numbers of nouns and verbs in the annotated data

summary(factor(abst.ann.df$upos))

## use the annotated df of the abstracts to build topic modeling at the document level

## See instructions at https://bnosac.github.io/udpipe/docs/doc6.html

## Build Latent Dirichlet Allocation model with nouns and verbs - see

## Fiona Martin and Mark Johnson. More Efﬁcient Topic Modelling Through a Noun Only Approach.

## Proceedings of Australasian Language Technology Association Workshop 2015.

dtf <- subset(abst.ann.df, upos %in% c("NOUN", "VERB"))

dtf$lemma=tolower(dtf$lemma) # fix instances we detected that the lemma of words are not in lower case

dtf1 <- document_term_frequencies(dtf, document = "doc_id", term = "lemma") # topic modeling is at the abstract level "doc_id"

## Create a document/term/matrix for building a topic model

lda.dtm <- document_term_matrix(x = dtf1)

dim(lda.dtm)

jterms=colnames(lda.dtm)

deleted.terms=grep("[-0-9<>/#%.=\\*\\?]",jterms,value=TRUE) # identify weird terms listed as nouns or verb in udpipe

lda.dtm1= dtm_remove_terms(lda.dtm, terms=deleted.terms)

jterms=colnames(lda.dtm1)

jterms.len = nchar(jterms)

jterms.short.terms=jterms.len > 3

deleted.terms=jterms[jterms.short.terms==FALSE]

lda.dtm2= dtm_remove_terms(lda.dtm1, terms=deleted.terms) # delete nouns or verbs with 1-3 characters with little meaning

## Remove nouns with low frequencies and remove abstracts without nouns or verbs

lda.dtm3 <- dtm_remove_lowfreq(lda.dtm2, minfreq = 5)

dim(lda.dtm)

dim(lda.dtm1)

dim(lda.dtm2)

dim(lda.dtm3)

head(dtm_colsums(lda.dtm3))

## identify removed abstracts through the steps above

jj = wkta$id %in% as.integer(rownames(lda.dtm3))

lda.deleted.abstract.ids=wkta$id[jj==FALSE] # abstracts with limited content are not used in ML training

length(lda.deleted.abstract.ids)

head(lda.deleted.abstract.ids)

lda.dtm=lda.dtm3

rm(lda.dtm1,lda.dtm2,lda.dtm3) # remove unused document-term matrices

# testing method="VEM" # Use this estimation method as the Bayesian method takes over 4 days without convergence (versus 14 hours for VEM)

lda.control.lst.test=list(verbose=5000) # this is more for the Bayesian method, now we use VEM method for estimation, so this is a place holder only

start.time <- Sys.time()

my.cluster <- makeCluster(spec=4, type = "SOCK") # SOCK stands for socket cluster, parallel processing

registerDoSNOW(my.cluster)

tm.lda.test <- LDA(x= lda.dtm, k = 300, method = "VEM", control = lda.control.lst.test) # set 300 topics

# Notes: the Bayesian approach to estimation ran for 4 days without convergence - we used the VEM estimation

stopCluster(my.cluster)

lda.time.test= Sys.time() - start.time # this step is time consuming, about 14 hours of run-time

# tm.lda <- readRDS("tm_lda.RData")

saveRDS(tm.lda.test, "scopingr_lda.RData")

## extract the posterior distributions of topics for each abstract in the corpus

sr.lda.X.test <- posterior(tm.lda.test)$topics

dim(sr.lda.X.test)

sr.lda.X=sr.lda.X.test # 300 topics

# saveRDS(sr.lda.X.test, "scopingr_lda_X.RData") #

# sr.lda.X.test <- readRDS("sr_lda_X_test.RData")

rm(sr.lda.X.test) # release memory -

##################################################################################################################################

## Steps 2-5. Build additional feature matrix and distance matrix to supplement those from Singular Value Decomposition (Figure 1)

## Clinical Concept Embeddings

## Run this section for Systematic review of clinical studies

## Step 2. Pre-process text - Annotate abstracts using the biomedical semantic annotator RysannMD

## Obtain Unique Medical Language System Clinical Concept Unique Identifiers UMLS CUI's for each abstract

## See John Cuzzola, Jelena Jovanovic, Ebrahim Bagheri. RysannMD: A biomedical semantic annotator balancing speed and accuracy.

## Journal of Biomedical Informatics 71 (2017) 91–109

## Step 3. Construct features as UMLS CUI's and obtain vector representations of CUI's using pre-trained word vectors for CUI's

## See Beam et al. Clinical Concept Embeddings Learned from Massive Sources of Multimodal Medical Data.

## Step 4. Obtain vector representations of abstracts as weighted average of vector representations of CUI's, weighting on CUI frequency

## Step 5. Calculate the distance between abstracts as the minimum amount of distance that the embedded CUI'ss of one abstract need to

## “travel” to reach the embedded CUI's of another abstract.

## Kusner M, Sun Y, Kolkin N, Weinberger K. From Word Embeddings To Document Distances.

## Proceedings of the 32 nd International Conference on Machine Learning, Lille, France, 2015.

######################################################################################################################################

## Step 2. Pre-process text - Annotate abstracts using the biomedical semantic annotator RysannMD

#### Create Dataset from the directory containing RysannMD outputs, each abstract is an annotated file

rysannmd_folder<-"/home/ba/ranking/rysannmd/MD.TA.all" # each abstract is a text file in this folder

rys_data <- data.frame(read_folder(rysannmd_folder)) %>% transmute(id, text)

# remove ".MD.title.abstract.txt" from id's in the first column of the dataset

rys_data[,1]=rys_data[,1] %>% map(sub,pattern=".MD.title.abstract.txt",replacement="") %>% as.integer()

# extract cui's and replace the text in each abstract in the dataset rys_data by a list of CUI's

start.time=Sys.time()

cui_dd <- rys_data %>% extr_cuis(certainty.threshold=0.5) # keep CUI's with high certainty of correct annotation - see function "extr_cuis" below

cui.time=Sys.time() - start.time # keep track of computing time

rm(rys_data)

## Step 3. Construct features as CUI's and obtain vector representations of CUI's using pre-trained CUI vectors from project cui2vec

## Create dtm

cui.dtm <- dfm(cui_dd$text, tolower = FALSE)

rownames(cui.dtm)=cui_dd$id

abstract.cuis=colnames(cui.dtm) # CUI's in abstracts

## Load the pre-trained vector representation of CUI from project cui2vec with 500 dimensions

cui2vec.file <- "/home/ba/ranking/csvfiles/cui2vec_pretrained.csv" # each row consists of a CUI and 500 variables

cui2vec.dd=read.csv(file=cui2vec.file,header=TRUE)

colnames(cui2vec.dd)[1]="cui" # name the column of CUI's as "cui"

## Match CUI's from the abstracts to CUI's from the pretrained dataset cui2vec

cuis.to.keep <- intersect(abstract.cuis, cui2vec.dd$cui)

## check the 'level' of matching

length(cuis.to.keep)/length(abstract.cuis)

## 44% of CUIs from our abstracts have their vectors in cui2vec - low coverage

## Create a new DTM that will keep only those common CUI's - TF

cui.dtm1 <- dfm_keep(cui.dtm, pattern=cuis.to.keep, valuetype="fixed", verbose=TRUE)

abstract.cuis=colnames(cui.dtm1) # CUI's in abstracts and in pre-trained cui2vec dataset

## Likewise, from cui2vec, select CUI's that are in the corpus abstracts

cuis.to.keep.indices <- which(cui2vec.dd$cui %in% cuis.to.keep) # rows in the cui2vec dataset

cui2vec.dd1=cui2vec.dd[cuis.to.keep.indices,]

dim(cui2vec.dd1)

## Order the columns in the cui2vec.dd1 dataset to be the same as in the columns of the cui.dtm1

jj = cui2vec.dd1[,-1] # drop the column containing CUI's names

jj = t(jj)

colnames(jj)=cui2vec.dd1$cui

jj = jj[,abstract.cuis] # ordering

jj[1:5,1:5] # check

abstract.cuis[1:5] # check

cui2vec.dd1=jj;

rm(jj);

## Step 4. Obtain vector representations of abstracts as weighted average of vector representations of CUI's, weighting on CUI frequency

start.time <- Sys.time()

sr.cui.X <- data.frame()

for(i in 1:nrow(cui.dtm1)) { # this is time consuming,about 10 hours

abst.tf <- as.matrix(cui.dtm1)[i,] # DTM

abst.matrix <- abst.tf * t(cui2vec.dd1) # weighted cloud of CUIs in 500d space

abst.mapped <- apply(abst.matrix, 2, mean) # the central point of the cloud

sr.cui.X <- as.data.frame(rbind(sr.cui.X, abst.mapped)) # store abstract representation in 500d

}

colnames(sr.cui.X) <- paste0("V",1:ncol(sr.cui.X)) # V1:V500

rownames(sr.cui.X) <- rownames(cui.dtm1) # abstract names

cui.time=Sys.time() - start.time # Time difference

sr.cui.X[1:5,1:5] # check

dim(sr.cui.X) #### matrix of n abstracts and 500 vectors

saveRDS(sr.cui.X, "sr_cui_X.RData")

# Load the saved object

# sr.cui.X <- readRDS("sr_cui_X.RData")

## Step 5. Calculate the distance between abstracts as the minimum amount of distance that the embedded words of one abstract need to

## “travel” to reach the embedded words of another abstract.

## Create a Relaxed Word Mover Distance (RWMD) object by specifying 2 input parameters:

## - word vector matrix with words given in rows and dimensions of the embedding space in columns; rows should have word names.

## - the method to be used for computing the distance between word vectors

cui.rwmd.model = RWMD$new(wv = t(cui2vec.dd1), method = "cosine") # Notes: transpose is needed here

## Now, we use the RWMD object and our DTM to compute WMD distances between

# each document pair. However, before that, we need to normalize TFs in

# the DTM matrix (required by the WMD algorithm; see the original paper)

start.time <- Sys.time() # this step is time consuming, about 23 hours

cui.dtm1.norm <- dfm_weight(cui.dtm1, scheme = "prop")

sr.cui.dist = dist2(x = cui.dtm1.norm, method = cui.rwmd.model, norm = 'none')

dim(sr.cui.dist) ##### WMD matrix for abstracts

cui.rwmd.time=Sys.time()-start.time

saveRDS(sr.cui.dist, "sr_cui_dist.RData")

# sr.cui.dist <- readRDS("sr_cui_dist.RData")

###############################################################################################################

## Steps 2-5. Build additional feature matrix and distance matrix to supplement those from Singular Value Decomposition (Figure 1)

## Word Embeddings: Global Vectors for Word Representation

## Run this section for Systematic review of non-clinical studies (e.g., SRs of health services research methods, SLR of computing topics)

## Step 2. Pre-process text to extract words from abstracts

## Step 3. Construct features as words and obtain vector representations of words using pre-trained word vectors from Glove

## Jeffrey Pennington, Richard Socher, Christopher D. Manning. GloVe: Global Vectors for Word Representation

## https://nlp.stanford.edu/projects/glove/

## Step 4. Obtain vector representations of abstracts as weighted average of vector representations of words, weighting on word frequency

## Step 5. Calculate the distance between abstracts as the minimum amount of distance that the embedded words of one abstract need to

## “travel” to reach the embedded words of another abstract.

## Kusner M, Sun Y, Kolkin N, Weinberger K. From Word Embeddings To Document Distances.

## Proceedings of the 32 nd International Conference on Machine Learning, Lille, France, 2015.

###############################################################################################################

## Step 2. Pre-process text to extract words from abstracts

glove.tokens <- tokens(x = wkta$text, what = "word", remove_numbers = TRUE,remove_punct = TRUE,

remove_symbols = TRUE, remove_hyphens= FALSE, ngrams=1) # words

## remove tokens with 1 or 2 characters only as they rarely bear any meaning

glove.tokens <- tokens_keep(x = glove.tokens, min_nchar = 3)

## to lower letter

glove.tokens <- tokens_tolower(glove.tokens)

## remove stopwords.

glove.tokens <- tokens_remove(glove.tokens, stopwords())

## Note that we are not stemming the tokens since words in the GloVe model were not stemmed, and we need to match against those words.

## Create DTM

glove.dtm <- dfm(glove.tokens, tolower=FALSE)

## Extract words (features) from the DTM since we need to match these against the words in the pre-trained GloVe model

abstract.words <- colnames(glove.dtm)

## ... and examine them

head(abstract.words, n = 20)

tail(abstract.words, n = 20)

## Notice the presence of words ending with "'s" (such as "kaiser's"). Replace such words with their version without "'s"

end.with.s <- str_detect(abstract.words, "(\\w+)'s$")

end.with.s <- abstract.words[which(end.with.s)]

words.no.s <- str_replace(end.with.s, "(\\w+)'s", "\\1")

## Replace, in the tokens object, tokens that end with "'s" with their 'cleaned' version

glove.tokens <- tokens_replace(glove.tokens, pattern = end.with.s, replacement = words.no.s)

## Step 3. Construct features as words and obtain vector representations of words using pre-trained word vectors from Glove

## Create again dtm

glove.dtm <- dfm(glove.tokens, tolower = FALSE)

## Create again a vector of vocabulary terms

abstract.words <- colnames(glove.dtm)

## Load the pre-trained GloVe word vectors of 840 billions terms and a vectorized space of 300 dimensions

glove.840B.300d.file <- "/home/ba/ranking/csvfiles/glove.840B.300d.txt"

start.time <- Sys.time()

g840B.300d <- scan(file = glove.840B.300d.file, what="", sep="\n")

glove.load.time=Sys.time() - start.time

## What we have read - g840B.300d - is in fact a huge character vector, consisting of millions of entries

## Each entry is given as a string that consists of 301 items delimited by a space:

## the 1st item is a word and the rest (300 items) are the estimated values of the 300 dimensions of that word

## Create a data frame out of the large vector read from the file

## (get_word_vectors_df() is defined in the UtilityFunctions.R script)

g840B.300d.df <- get_word_vectors_df(g840B.300d, verbose = TRUE)

dim(g840B.300d.df)

# save this large object

# saveRDS(g840B.300d.df, "g840B_300d_df.RData")

# Load the saved object

# g840B.300d.df <- readRDS("g840B_300d_df.RData")

## Remove unused objects to release memory

remove(g840B.300d)

## Take the words from the GloVe model - we need these words to match them against the features (words) from the corpus DTM

glove.words <- colnames(g840B.300d.df)

## Match words from the abstracts to words from Glove

words.to.keep <- intersect(abstract.words, glove.words)

## check the 'level' of matching

length(words.to.keep)/length(abstract.words)

## 71% of words from our DTM have their vectors in GloVe

## Let's briefly inspect words from abstracts that are not in GloVe

setdiff(abstract.words, glove.words)[1:50]

## 30% missing of words, mainly words pertaining to KS methods

## Create a new DTM that will keep only those words (columns) - TF

glove.dtm1 <- dfm_keep(glove.dtm, pattern=words.to.keep, valuetype="fixed", verbose=TRUE)

## Likewise, from GloVe, select word vectors that will be used for building a feature set, that is, words present in abstracts

glove.to.keep.indices <- which(glove.words %in% words.to.keep)

g840B.300d.df1 <- g840B.300d.df[,glove.to.keep.indices]

## Order the columns (words) in the reduced g840B_300d_df1, to be the same as in the reduced glove_dtm1

g840B.300d.df1 <- g840B.300d.df1[,colnames(glove.dtm1)]

## Before proceeding, remove large objects that are no longer needed

remove(g840B.300d.df, glove.tokens, glove.words, abstract.words, glove.dtm)

## Step 4. Obtain vector representations of abstracts as weighted average of vector representations of words, weighting on word frequency

## Compute feature values for each abstract as the (coordinate-wise) TF-weighted mean value across all the word vectors.

##

## Note that after the above reduction of DTM and GloVe to the common set of

## features (words), the two matrices have the same number of columns.

## Now, we will take each abstract (row) from the DTM and multiply it with the transposed

## GloVe matrix, thus, in fact weighting word vectors in GloVe with the post-specific

## TF weights of the corresponding words. As the result, we will get a matrix of

## TF-weighted word vectors (words in rows, dimensions in columns) for each abstract.

## Next, we take the mean value (across words) for each dimension (columns), to obtain

## a new feature vector for each abstract; these vectors have the same number of features

## as there are dimensions in the GloVe model (300). This way, we are, in fact,

## translating the existing feature space (words in DTM) into a new feature space

## (dimensions of the GloVe word vectors).

start.time <- Sys.time() # this step is time consuming

sr.glove.X <- data.frame()

for(i in 1:nrow(glove.dtm1)) {

abst.tf <- as.matrix(glove.dtm1)[i,] # DTM

abst.matrix <- abst.tf * t(g840B.300d.df1) # weighted cloud of words in 300d space

abst.mapped <- apply(abst.matrix, 2, mean) # the central point of the cloud

sr.glove.X <- as.data.frame(rbind(sr.glove.X, abst.mapped)) # store document representation in 300d

}

colnames(sr.glove.X) <- paste0("V",1:ncol(sr.glove.X))

dim(sr.glove.X) #### matrix of n abstracts and 300 vectors

glove.time=Sys.time() - start.time # Time difference of 4.600928 mins

saveRDS(sr.glove.X, "scopingr_glove_X.RData")

# Load the saved object

# sr.glove.X <- readRDS("sr_glove_X.RData") #

## Step 5. Calculate the distance between abstracts as the minimum amount of distance that the embedded words of one abstract need to

## “travel” to reach the embedded words of another abstract.

## Create a Relaxed Word Mover Distance (RWMD) object by specifying 2 input parameters:

## - word vector matrix with words given in rows and dimensions of the embedding space in columns; rows should have word names.

## - the method to be used for computing the distance between word vectors

rwmd.model = RWMD$new(wv = t(g840B.300d.df1), method = "cosine")

## Now, we use the RWMD object and our DTM to compute WMD distances between

# each document pair. However, before that, we need to normalize TFs in

# the DTM matrix (required by the WMD algorithm; see the original paper)

start.time <- Sys.time() # this step is time consuming

glove.dtm1.norm <- dfm_weight(glove.dtm1, scheme = "prop")

sr.glove.dist = dist2(x = glove.dtm1.norm, method = rwmd.model, norm = 'none')

dim(sr.glove.dist) ##### WMD matrix for abstracts

glove.rwmd.time=Sys.time()-start.time

# saveRDS(sr.glove.dist, "scopingr_glove_dist.RData")

#############################################################

# Step 5. Quantify citation similarity (Figure 1)

#############################################################

start.time <- Sys.time()

my.cluster <- makeCluster(spec=6, type = "SOCK") # parallel processing

registerDoSNOW(my.cluster)

sr.svd.dist = dist2(x=sr.svd.X, method="cosine") # symmetric matrix of pairwise distances between pairs of citations # SA - Threshold - Repeat

#sr.lda.dist = dist2(x=sr.lda.X, method="cosine")

stopCluster(my.cluster)

Sys.time() - start.time

dim(sr.svd.dist) # Distance matrix to be used for candidate selection for screening in the workflow steps

dim(sr.lda.dist)

##################################################################################

# Workflow function - Phases 1 and 2 (Figure 1)

##################################################################################

## inputs: (see Table 2 in the main paper)

## m.x - Document-feature matrix from SVD

## m.distance: List of 3 matrices of dimension n x n of pairwise distances between n abstracts: svd, lda, and text2vec

## dd - dataset with columns "id" and "status" of abstracts. Specify status categories in pos.label and neg.label variables below.

## l.seeds: initial list of ID's of seed studies for prioritizing abstracts for screening by human reviewers

## n.rounds: maximum number of rounds of screening by human reviewers (e.g., 20 rounds, see Table 2 in the paper)

## n.initial: Minimum sample size of the initial train dataset, such as 600 abstracts when the corpus of abstracts is represented by 300 features (see Table 2)

## pick.init: The size k of k nearest-neighbors of an eligible abstract used to build the initial train dataset (e.g., k=8, see Table 2)

## pick.ml: The size k of k nearest-neighbors of an eligible abstract used to iterate the loop in the Workflow Phase 2 (e.g., k=15, see Table 2)

## outputs: (see Table 2A in the online Appendix)

## ll.candidates: list of lists of rows of abstracts in the corpus to be screened by human reviewers

## d.results: Step-specific results of the execution of the workflow (see examples in the Appendix of the paper)

## rf.svd: final random forest model with feature representations from singular value decomposition SVD

workflow.phase1and2 <- function(m.x, m.distance, dd, l.seeds, n.rounds, n.initial=600, pick.init=8, pick.ml=15) {

system("echo \"Initialization\" | mailx -s \'Workflow starts \' ba.pham@theta.utoronto.ca") # tracking workflow steps over some running hours

## initialization

ll.candidates=NULL # list of lists of candidate abstracts for screening by human reviewers

d.results= data.frame(matrix(NA,nrow=4*n.rounds,ncol=14)) # panel data to record step-specific results from the workflow history

colnames(d.results)=c("phase","round","n.seeds","n.candidates","n.eligibles","percent",

"precision","recall","f1","accuracy","tp","fp","fn","tn") # see Table 2A of online Appendix

cum.candidate.rows=NULL # row indices of abstracts that have been screened by human reviewers. The indices refer to the rows of the corpus dataset

initial.phase.done = FALSE # flag to indicate the initial phase that accumulates the training dataset is done

train.level.done=FALSE # flag to indicate the two iterations of ML phase is done

metric="Sens" # metric to maximize in cross-validation of ML models: 1) maximizing sensitivity for all cross-validated models and 2) maximizing ROC for the final model

curr.eligibles=unlist(lapply(l.seeds,indx.lkup.all,dd=dd)) # look up the rows in the corpus of the initially eligible abstracts, see function "indx.lkup.all" below

cum.eligibles.rows=NULL # cumulative list of all seeds across iterations

c.round = 1 # round denotes the number of times the workflow interacts with human reviewers for abstract screening

while(c.round<n.rounds) { # rounds denote the number of times the workflow asks human reviewers to screen batches of selected abstracts

if(!initial.phase.done) { # Start Phase 1 to gather the training data by iterative steps 6-9, Figure 1)

## Step 6 - Prioritize citations using 3 distance matrices: SVD, feature embeddings and topic modeling LDA,(Figure 1) ##################

curr.candidate.rows = similar.abstracts(distance.matrices=m.distance,seed.id=curr.eligibles,pick=pick.init) # Step 6 - identify abstracts similar to an eligible abstract

curr.candidate.rows=c(curr.candidate.rows,curr.eligibles) # include the seeds into current candidates

curr.candidate.rows=unique(curr.candidate.rows) # remove duplicates

duplicates = curr.candidate.rows %in% cum.candidate.rows # identify duplications with already screened abstracts

curr.candidate.rows =curr.candidate.rows[duplicates==FALSE] # only candidates that are yet to be screened

if(length(curr.candidate.rows)==0) {

cat("Initial phase to generate training data: cannot generate new candidates \n")

initial.phase.done=TRUE

} # no new candidates

else {

cum.candidate.rows=c(cum.candidate.rows,curr.candidate.rows) # update the list of all candidates

cum.candidate.rows=unique(cum.candidate.rows) # remove duplicates

cum.candidate.rows=na.omit(cum.candidate.rows) # remove missing row numbers, if necessary

cum.eligibles.rows=c(cum.eligibles.rows,curr.eligibles) # update the list of all eligible abstracts

cum.eligibles.rows=unique(cum.eligibles.rows) # remove duplicates

## recording step-specific results of steps 6-7 (Figure 1)

n.seeds=length(curr.eligibles) # record the number of eligible abstracts in this round of human screening

curr.status=dd$status[cum.candidate.rows] # results of screening by human reviewers

numerator.prevalence=length(cum.candidate.rows[curr.status==pos.label]) # record the number of predicted eligible abstracts

denomerator.prevalence=length(cum.candidate.rows) # record the number of screened abstracts

recording(d.results,"initial",c.round,c.round,n.seeds,denomerator.prevalence,numerator.prevalence,

rep(NA,4),rep(NA,4)) # recording the step-specific results into the workflow panel data, see sample of the panel in the Appendix

## Step 7 - Screen citations (Figure 1) #####################

ll.candidates[[c.round]]=curr.candidate.rows # record the set of abstracts to be screened by human reviewers

curr.status=dd$status[curr.candidate.rows] # results of screening by human reviewers

curr.eligibles=curr.candidate.rows[curr.status==pos.label] # identify eligible citations as seeds for the next round of iteration

duplicates=curr.eligibles %in% cum.eligibles.rows # identify duplications in the cumulative list of eligible abstracts

curr.eligibles=curr.eligibles[duplicates==FALSE] # Step 9 - remove duplicates. Newly identified eligible abstracts are used in the next iteration of steps 6-9

if(length(curr.eligibles)==0) {

cat("Initial phase to generate training data: cannot generate new seeds \n")

initial.phase.done=TRUE

} # no new eligible abstracts

if(length(cum.candidate.rows)>n.initial) { # step 8 (Figure 1)

cat("Initial phase to generate training data: completed \n")

initial.phase.done=TRUE

k=c.round+1 # set up for fitting ML models - index to the row of the workflow panel

} # accumulate enough training data

}

## prepare for another round of human screening, if needed

c.round=c.round+1

} # end if(!initial.phase.done)

if(initial.phase.done && !train.level.done) { # start the Workflow Phase 2 in Figure 1 ##################

system("echo \"ML modeling\" | mailx -s \'Workflow in ML phase \' ba.pham@theta.utoronto.ca") # tracking workflow steps over some running hours

## Step 7. Screen citations by human reviewers (Figure 1) ######################################

curr.dd = dd[cum.candidate.rows,] # current training dataset, given the screening results of the list of cumulative candidates

### collect step-specific statistics from the training dataset

numerator.prevalence=length(curr.dd$status[curr.dd$status==pos.label]) # record the number of eligible abstracts

denomerator.prevalence=nrow(curr.dd) # record the number of screened abstracts in the training dataset

## Step 10. Assemble training data (Figure 1) ####################################################

curr.m.x=m.x[cum.candidate.rows,] # assemble the X matrix of features from the SVD method

curr.m.xy=data.frame(curr.m.x) %>% mutate(status=curr.dd$status) # assemble training dataset, including features and screening results

## Step 11. Training random forest models (Figure 1) #################################################

rf.svd=rf.model(mdata=curr.m.xy, metric=metric)

## Step 12. Predict eligible abstracts (Figure 1) #################################################

pred.rf.raw.svd.corpus <- predict(rf.svd, newdata = m.x, type="raw") # corpus level

rf.confusion.m <- confusionMatrix(data = pred.rf.raw.svd.corpus, reference = dd$status, positive=pos.label) # evaluate model performance

m.rf.eval <- get_eval_measures(rf.confusion.m) # calculate performance measures

jtp = rf.confusion.m$table

m.rf.abcd <- c(jtp[1,1],jtp[1,2],jtp[2,1],jtp[2,2]) # Extract TP, FP, FN, TN

### recording the results of the RF classifier into workflow panel data

m.round=d.results[k-1,"round"]+2 # each fitted RF involves 2 sets of candidates for screening - see below

recording(d.results,"rf.svd",k,m.round,NA,denomerator.prevalence,numerator.prevalence, m.rf.eval,m.rf.abcd)

k= k + 1 # next rows

cat("fitting rf.svd \n")

## Step 12 - Prepare predicted eligible abstracts for screening (Figure 1) #####################

curr.candidates.rows = which(pred.rf.raw.svd.corpus == pos.label) # identify rows with predicted eligibles in the corpus

duplicates=curr.candidates.rows %in% cum.candidate.rows # identify duplications with already screened abstracts

curr.candidate.rows = curr.candidate.rows[duplicates==FALSE] # remove duplicates

## Step 12. Do we have predicted eligible abstracts to be screened by human reviewers?

if(length(curr.candidate.rows)==0) {

train.level.done = TRUE # iterate until no predicted eligible abstracts are possible

c.round = n.rounds # no more iteration

}

else {

ll.candidates.index=length(ll.candidates) + 1 # Increment the list of screened abstracts

ll.candidates[[ll.candidates.index]]=curr.candidate.rows # Step 6 - record the set of predicted eligible abtracts to be screened by human reviewers

# Step 7 - use of the screened results to identify eligible abstracts and look for similar abstracts to those newly identified eligible abstracts

curr.dd=dd[curr.candidates.rows,]

j.status=curr.dd$status # get the screened results from the screening candidates

j.eligibles <- j.status == pos.label # identify eligible abstracts among the screening candidates

j.id <- curr.dd$id[j.eligibles == TRUE] # obtain abstract ID's of the newly identified eligible abstracts

curr.eligibles=which(dd$id %in% j.id) # look up the rows in the corpus - this set becomes the subjects for the next iteration of steps 10-14 and 6-7

duplicates=curr.eligibles %in% cum.eligibles.rows # identify duplications in eligible abstracts

curr.eligibles=curr.eligibles[duplicates==FALSE] # remove duplicates

if(length(curr.eligibles)==0) { # Step 14 - Can we still identify newly identified eligible abstracts?

train.level.done = TRUE # iterate until no new eligible abstracts are possible

c.round = n.rounds # no more iteration

}

else {

# update the list of screened abstracts

cum.candidate.rows=c(cum.candidate.rows,curr.candidate.rows) # update the list of all candidates in terms of rows in the corpus dataset

cum.candidate.rows=unique(cum.candidate.rows) # remove duplicates

cum.candidate.rows=na.omit(cum.candidate.rows) # remove missing row numbers

cum.eligibles.rows=c(cum.eligibles.rows,curr.eligibles) # update the list of all eligibles that have been identified

cum.eligibles.rows=unique(cum.eligibles.rows) # remove duplicates

# Step 6 - Identify abstracts similar to the newly identified eligibles

curr.candidate.rows = similar.abstracts(distance.matrices=m.distance,seed.id=curr.eligibles,pick=pick.ml) # identify abstracts similar to newly identified eligibles

curr.candidate.rows=c(curr.candidate.rows,curr.eligibles) # include the seeds into current candidates

curr.candidate.rows=unique(curr.candidate.rows) # remove duplicates

cum.candidate.rows=na.omit(cum.candidate.rows) # remove missing row numbers

duplicates = curr.candidate.rows %in% cum.candidate.rows # identify duplications with already screened citations

curr.candidate.rows=curr.candidate.rows[duplicates==FALSE] # remove duplicates

## Step 7 - abstracts to be manually screened

ll.candidates.index=length(ll.candidates) + 1 # index to the next set of abstracts for screening by human reviewers

ll.candidates[[ll.candidates.index]]=curr.candidate.rows # record the set of abtracts to be screened by human reviewers

## prepare for another round of human screening, if needed

c.round=c.round+1 # each Workflow Phase 2 involves two rounds of manual screening

# Step 10 - Prepare the screening results for updating the training dataset

curr.dd=dd[curr.candidates.rows,]

j.status=curr.dd$status # get the screened results from the screening candidates

j.eligibles <- j.status == pos.label # identify eligible abstracts among the screening candidates

j.id <- curr.dd$id[j.eligibles == TRUE] # obtain abstract ID's of predicted eligibles

curr.eligibles=which(dd$id %in% j.id) # look up the rows in the corpus dataframe - this set becomes the new seeds

duplicates=curr.eligibles %in% cum.eligibles.rows # identify duplications in seeds

curr.eligibles=curr.eligibles[duplicates==FALSE] # remove duplicates

# Step 10 - Update the list of screened abstracts and the list of eligible abstracts identified up to this point

cum.candidate.rows=c(cum.candidate.rows,curr.candidate.rows) # update the list of all candidates in terms of rows in the corpus dataset

cum.candidate.rows=unique(cum.candidate.rows) # remove duplicates

cum.candidate.rows=na.omit(cum.candidate.rows) # remove missing row numbers

cum.eligibles.rows=c(cum.eligibles.rows,curr.eligibles) # update the list of all eligibles that have been identified

cum.eligibles.rows=unique(cum.eligibles.rows) # remove duplicates

## prepare for another round of human screening, if needed

c.round=c.round+1 # each ML iteration involves two rounds of manual screening

} # end if(length(curr.eligibles)==0)

} # end of if(length(curr.candidate.rows)==0)

} # end if(initial.phase.done && !train.level.done)

} # end while(c.round<n.rounds)

# tracking messages

if(!initial.phase.done) {cat("Initial phase to generate training data: Not completed \n")}

if(!train.level.done) {cat("ML phase - training level: Not completed \n")}

d.results=d.results %>% dplyr::filter(!is.na(round)) # remove blank rows in the workflow panel data

output=list(candidates=ll.candidates, results=d.results, rf.svd=rf.svd) # compile outputs, the final prediction model is the Random Forest with SVD feature representation

return(output)

} # end function

### Functions called by the Workflow functions

## select abstracts similar to the seed abstracts for screening, given a distance matrix

## return the rows of abstracts in the corpus dataset for human screening

## requires a distance matrix, list of seed studies and the number k fo the k-nearest neighbors

similar.abstracts.one = function(distance.matrix=NULL,seed.id=seed.id,pick=25) {

# cat("In similar abstracts - seed.id: ",seed.id,"\n" )

m.colnames=colnames(distance.matrix) # row and column names of the distance matrix must be sequenced from 1 to number of abstracts

list.pick=NULL

nn=length(seed.id)

pick = pick + 1 # skip the first one, take from 2 to pick + 1, as the abstracts that are closest to the seed

for(i in c(1:nn)) {

jtemp = distance.matrix[seed.id[i],] # take distances of each seed abstract relative to others

jord=order(jtemp,decreasing=FALSE) # line up the more similar abstracts to the seed

jextract.colnames=m.colnames[jord]

current.pick=jextract.colnames[2:pick]

list.pick=c(list.pick,current.pick)

}

list.pick=unique(list.pick)

list.pick=as.integer(list.pick)

return(list.pick) # return a list of rows in the main corpus database

}

## select abstracts similar to the seed abstracts for screening, given "n" distance matrices

similar.abstracts = function(distance.matrices=distance.matrices,seed.id=seed.id,pick=25) {

nn = length(distance.matrices) # expected distance matrices from SVD, LDA and feature embeddings

list.pick=NULL # list of candidate abstracts for human screening

for (i in c(1:nn)) {

curr.matrix=distance.matrices[[i]]

curr.pick = similar.abstracts.one(distance.matrix=curr.matrix,seed.id=seed.id,pick=pick)

# cat("in here ", i, "candidates", curr.pick, "\n")

list.pick=c(list.pick,curr.pick)

}

return(unique(list.pick)) # return a list of rows in the main corpus database

}

## look up the row of an abstract ID

indx.lkup.all=function(x,dd) {which(dd$id==x)} # look up the row of an abstract ID from the input dataframe

### Fit the Random forest model through Cross-Validation (CV)

### Input data is a dataframe with 300 predictors, the last column is labeled as "status", denoting the screening results, and is the response variable

### Output is the fitted RF model - Specify "ROC" or "Sens"

rf.model <- function(mdata, metric="ROC") { # or metric="Sens"

cv.cntrl.rf <- trainControl(method = "cv", number = 6, sampling='smote', search = "grid",

summaryFunction=twoClassSummary, classProbs = TRUE,verboseIter=FALSE) # set up cross-validation parameters, see package 'caret'

max.n.leaves = as.integer(ncol(mdata)/10) # preferrably a number much smaller than the number of features such as 300 for svd and lda or 500 for text2vec

rf.grid <- expand.grid(mtry = as.integer(seq(from = 1, to = max.n.leaves, length.out = 20))) # possible values for the # of predictors of decision trees in the RF

set.seed(seed) # fix value for the stream of pseudo-random number generator

## Create a cluster to work on logical cores;

assign("last.warning", NULL, envir = baseenv()) # clear messages

unregister() # clear any remaining registered data on parallel processing

my.cluster <- makeCluster(spec=6, type = "SOCK") # specify the number of clusters for parallel processing

registerDoSNOW(my.cluster) # start the parallel processing

m.rf.cv <- train(status ~ ., data=mdata, method = "rf", ntree=500, metric=metric,

tuneGrid = rf.grid, trControl=cv.cntrl.rf, maximize=TRUE) # fit the RF model through CV

stopCluster(my.cluster) # end parallel processing

assign("last.warning", NULL, envir = baseenv()) # clear messages

unregister() # clear any remaining registered data on parallel processing

return(m.rf.cv)

}

## Recording specific results of the workflow in a dataframe (see Table 2A for sample output)

recording = function(d.results,phase,k,round,n.seeds,n.candidates,n.eligibles,eval,table) {

jj=d.results # obtain a copy of the current dataframe of step-specific results

jj[k,"phase"]=phase # worflow phases: initial phase and ML phase, which specifies RF, SVM, Ensemble of the two

jj[k,"round"]=round # the number of times the Workflow function interacts with human reviewers to help screening citations for the training dataset

jj[k,"n.seeds"]=n.seeds # number of seeds used in the near-neighboring procedure to identify candidates for screening by human reviewers

jj[k,"n.candidates"]=n.candidates # number of candidate citations required screening by human reviewers

jj[k,"n.eligibles"]=n.eligibles # number of eligible citations from the current round of screening

jj[k,"percent"]=round(100*n.eligibles/n.candidates) # percent eligible citation from the current round of screening

jj[k,c("precision","recall","f1","accuracy")]=eval # RF or SVM performance measures

jj[k,c("tp","fp","fn","tn")]=table # breakdown of cells in the 2x2 tables

eval.parent(substitute(d.results<-jj)) # call by reference https://www.r-bloggers.com/call-by-reference-in-r/ to put the updated results in the d.results

}

### The function extracts some basic evaluation metrics from the model evaluation object

### produced by the confusionMatrix() function of the caret package

get_eval_measures <- function(model_eval) {

metrics <- c("Precision", "Recall", "F1", "Accuracy")

eval_measures <- model_eval$byClass[metrics[1:3]]

eval_measures <- c(eval_measures, model_eval$overall[metrics[4]])

eval_measures

}

### Ensemble classification with positive is identified if at least one classifier is positive

ensemble23 = function(l1,l2,l3=NULL,positives=pos.label,negatives=neg.label) { # l1, l2, l3 are the raw predictions from the classifiers

jout = rep(negatives,length(l1)) # "INCLUDE" if either classifier labeled as "INCLUDE"

ja=l1 == positives # e.g., RF

jb=l2 == positives # SVM

if(length(l3)==0) {

jc=ja | jb

}

else {

jc=l3 == positives # XGBT

jc=ja|jb|jc

}

jout[jc]=positives # if any classifier is "INCLUDE"

jout=factor(jout,levels=c(positives,negatives))

return(jout)

}

## function to convert uncertainty values (some in scientific format e.g., 0.76E-4) into numeric values

scientific_format_to_decimal=function(x) {

indx=regexpr("[E]",x,ignore.case=TRUE)[1]

if(indx>=1) {

num=substr(x,start=1,stop=indx-1);

n.exp=substr(x,start=indx+1, stop=nchar(x));

y=as.numeric(num)*10^as.numeric(n.exp);

} else y=as.numeric(x)

return(y)

}

### Extract Concept Unique Identifiers (CUI) from the output of the Annotator RysannMD for each abstract

### See John Cuzzola, Jelena Jovanovic, Ebrahim Bagheri. RysannMD: A biomedical semantic annotator balancing speed and accuracy.

### Journal of Biomedical Informatics 71 (2017) 91–109

### Input: annotated text from RysannMD and a threshold of uncertainty

extr_cuis <- function(rys_data,certainty.threshold=0.5) {

### UMLS CUI coding pattern: UMLS(cui)\":\"C0035647;

cui.pattern="UMLS\\(cui\\)\\\":\\\"C[0-9]{7}" # search patterns - noted the "\\(" for the escape of the special character "("

partial.cui.pattern=16 # number of trailing characters that need to be removed after matching pattern

### Uncertainty estimate of CUI - Pattern: "uncertainty\":6.1185281268738E-5

uncertainty.pattern="uncertainty\\\":[0-9\\.E-]*"

partial.uncertainty.pattern=14

op=rys_data

for(i in c(1:nrow(op))) {

# extract cui's

abst.cuis= rys_data$text[i] %>% str_extract_all(cui.pattern) %>% unlist() %>% str_remove(substr(cui.pattern,start=1,stop=partial.cui.pattern))

len1=length(abst.cuis)

# extract uncertainty and convert to numeric using a procedure in the UtilityFunctions.R

abst.uncertainty=rys_data$text[i] %>% str_extract_all(uncertainty.pattern) %>% unlist() %>% str_remove(substr(uncertainty.pattern,start=1,stop=partial.uncertainty.pattern))

len2=length(abst.uncertainty)

uncertainty=abst.uncertainty %>% map(scientific_format_to_decimal) %>% unlist()

certainty = 1-uncertainty # vector of certainty estimates

if(len1!=len2) {

cat("Not matching for list of CUI's and list of uncertainty estimates for abstract: ",i,"\n")

absts="NA"

} else {

jindx=certainty>=certainty.threshold

absts=abst.cuis[jindx==TRUE] %>% str_c(collapse=" ") # only kept the cui's with high certainty

}

op$text[i]= absts

}

return(op)

}

## The function creates a data frame out of the word vectors

## that originate from a pre-trained GloVe model (m_glove)

get_word_vectors_df <- function(m_glove, verbose = FALSE) {

# initialize space for values and the names of each word in the model

n_words <- length(m_glove)

vals <- list()

names <- character(n_words)

# loop through to gather values and names of each word

for(i in 1:n_words) {

if (verbose) {

if(i %% 5000 == 0) {print(i)}

}

this_vec <- m_glove[i]

this_vec_unlisted <- unlist(strsplit(this_vec, " "))

this_vec_values <- as.numeric(this_vec_unlisted[-1])

this_vec_name <- this_vec_unlisted[1]

vals[[i]] <- this_vec_values

names[i] <- this_vec_name

}

# convert the list to a data frame and attach the names

glove_df <- data.frame(vals)

names(glove_df) <- names

glove_df

}

## The function computes harmonic mean for the given input vector

harmonicMean <- function(values, precision=2000L) {

require("Rmpfr")

valMed <- median(values)

as.double(valMed - log(mean(exp(-mpfr(values, prec = precision) + valMed))))

}

## The function reads all files from the given folder (infolder)

## into a data frame and returns the created data frame

read_folder <- function(infolder) {

data_frame(file = dir(infolder, full.names = TRUE)) %>%

mutate(text = map(file, read_lines)) %>%

transmute(id = basename(file), text) %>%

unnest(text) # text is a list-column; unnest transforms each element of the list into a row

}

# clearance of previous parallel cluster data in the system

# https://stackoverflow.com/questions/25097729/un-register-a-doparallel-cluster

unregister <- function() {

env <- foreach:::.foreachGlobals

rm(list=ls(name=env), pos=env)

}

### End of the Workflow algorithm

# Set up for workflow execution -

# NOTES: These steps are messy right now because they were modified as I tested things out. It will be a few lines once the testing is done

# abstracts with limited content are not used in ML training, for SR type 1 diabetes, n=68

dim(wkta) # 16375 x 5, 16375 - 68 = 16307

length(lda.deleted.abstract.ids)

lda.deleted.abstract.rows=unlist(lapply(lda.deleted.abstract.ids,indx.lkup.all,dd=wkta)) # look up the rows of the deleted abstract ID's

length(lda.deleted.abstract.rows)

# corpus data frame

jj.dd = wkta %>% dplyr::select(id, abstract, status)

dim(jj.dd) # 16375 x 3

jj.dd1= jj.dd[-lda.deleted.abstract.rows,]

dim(jj.dd1) # 16307 x 3

# X matrices

dim(sr.svd.X) # 16375 x 300

length(wkta$id) # matching 16375

sr.svd.X1=sr.svd.X[-lda.deleted.abstract.rows,] # SA - Threshold - Repeat

dim(sr.svd.X1) # 16307 x 300

sr.lda.X1=sr.lda.X # LDA

dim(sr.lda.X1) # 16307 x 300

# Scoping review of KS Methods

dim(sr.glove.X)

sr.glove.X1=sr.glove.X[-lda.deleted.abstract.rows,]

dim(sr.glove.X1)

# This is for SR T1D only

dim(sr.cui.X) # 16376 500

j.sr.cui.X.ids=as.integer(rownames(sr.cui.X))

jjone=j.sr.cui.X.ids %in% wkta$id

summary(jjone)

j.sr.cui.X.ids[jjone==FALSE] # ID is 662239

which(j.sr.cui.X.ids==662239) # row 178

jj.sr.cui.X=sr.cui.X[-178,]

jj.guest=as.integer(rownames(jj.sr.cui.X))==wkta$id

summary(jj.guest) # equal

sr.cui.X.old=sr.cui.X

sr.cui.X=jj.sr.cui.X # 16375 x 500

sr.cui.X1=sr.cui.X[-lda.deleted.abstract.rows,]

dim(sr.cui.X1) # 16307 x 500

# distance matrices

dim(sr.svd.dist) # 16375 16375 # SA - Threshold - Repeat

sr.svd.dist1=sr.svd.dist[-lda.deleted.abstract.rows,-lda.deleted.abstract.rows]

dim(sr.svd.dist1) # 16307 x 16307

# sr.lda.dist1 = dist2(x=sr.lda.X1, method="cosine")

dim(sr.lda.dist)

sr.lda.dist1=sr.lda.dist

dim(sr.lda.dist1) # 16307 16307

sr.glove.dist1=sr.glove.dist[-lda.deleted.abstract.rows,-lda.deleted.abstract.rows] # Scoping review of KS Methods

dim(sr.glove.dist1)

# SR of T1D only

dim(sr.cui.dist) # 16376 x 16376

sr.cui.dist.old=sr.cui.dist

sr.cui.dist=sr.cui.dist[-178,-178]

dim(sr.cui.dist) # 16375 16375

sr.cui.dist1=sr.cui.dist[-lda.deleted.abstract.rows,-lda.deleted.abstract.rows]

dim(sr.cui.dist1) # 16307 x 16307 - this is Word Mover Distance

# remove titles only

jtitles.only=which(jj.dd1$abstract=="") # remove rows of titles only

length(jtitles.only) # 1993 titles only

# corpus data frame

jj.dd2=jj.dd1[-jtitles.only,c(1,3)]

dim(jj.dd2) # 14314 x 2

# X matrices

sr.svd.X2=sr.svd.X1[-jtitles.only,] # SA - Threshold - Repeat

dim(sr.svd.X2) # 14314 x 300

sr.lda.X2=sr.lda.X1[-jtitles.only,]

dim(sr.svd.X2) # 14314 x 300

sr.cui.X1=sr.glove.X1 # SR T1D and Scoping of KS Methods

sr.cui.X2=sr.cui.X1[-jtitles.only,]

dim(sr.cui.X2) # 14314 x 500

# distance matrices

sr.svd.dist2=sr.svd.dist1[-jtitles.only,-jtitles.only] # SA - Threshold - Repeat

dim(sr.svd.dist2) # 14314 x 14314

sr.lda.dist2=sr.lda.dist1[-jtitles.only,-jtitles.only]

dim(sr.lda.dist2) # 14314 x 14314

sr.cui.dist1=sr.glove.dist1

sr.cui.dist2=sr.cui.dist1[-jtitles.only,-jtitles.only]

dim(sr.cui.dist2) # 14314 x 14314

## Set the row and column names to the sequential order of how the abstracts were arranged in the input file

rownames(sr.svd.dist2)=colnames(sr.svd.dist2)=seq(from=1, to=nrow(sr.svd.dist2),by=1) # SA - Threshold - Repeat

rownames(sr.lda.dist2)=colnames(sr.lda.dist2)=seq(from=1, to=nrow(sr.lda.dist2),by=1)

rownames(sr.cui.dist2)=colnames(sr.cui.dist2)=seq(from=1, to=nrow(sr.cui.dist2),by=1)

## list of seed abstracts for SR T1D

seed.abstract.ids= c(100000,678127,664273,667053,662836)

jj.seeds.rows=unlist(lapply(seed.abstract.ids,indx.lkup.all,dd=jj.dd2)) # look up the rows of the abstract ID's

## list of seed abstracts for Scoping Review of KS Methods

seed.abstract.ids= c(10000,48803,45265,47160)

jj.seeds.rows=unlist(lapply(seed.abstract.ids,indx.lkup.all,dd=jj.dd2)) # look up the rows of the abstract ID's

## lists of matrices of X and distances

colnames(sr.svd.X2) <- paste0("V",1:ncol(sr.svd.X2)) # SA - Threshold - Repeat

colnames(sr.lda.X2) <- paste0("V",1:ncol(sr.lda.X2))

colnames(sr.cui.X2) <- paste0("V",1:ncol(sr.cui.X2))

j.m.x=list(svd=sr.svd.X2, text2vec=sr.cui.X2) # use 2 feature representations # SA - Threshold - Repeat

# j.m.x=list(svd=sr.svd.X2, text2vec=sr.cui.X2, lda=sr.lda.X2) # use 3 feature representations

j.m.distance=list(svd=sr.svd.dist2,lda=sr.lda.dist2,text2vec=sr.cui.dist2) # SA - Threshold - Repeat

j.m.distance1=list(svd=sr.svd.dist2,text2vec=sr.cui.dist2)

##

## Main analysis

start.time.wf <- Sys.time()

wf.test=workflow.phase1and2(m.x=sr.svd.X2, m.distance=j.m.distance,dd=jj.dd2,l.seeds=seed.abstract.ids,n.rounds=20,n.initial=600,pick.init=8,pick.ml=15)

wf.time12=Sys.time() - start.time.wf

#

# write.csv(wf.test$results,file="phases12-sr1.csv")

# write.csv(wf.test$results,file="phases12-scoping1.csv")
